# Intrinsically Bioactive Tannic Acid-Grafted Succinoglycan for Self-Healing and Stimuli-Responsive Drug Delivery Hydrogels

**DOI:** 10.3390/gels12090828

**Published:** 2026-09-10

**Authors:** Sang-Il Park, Kyungho Kim, Sungmin Rhyu, Seunho Jung

**Affiliations:** 1 Department of Bioscience and Biotechnology, Microbial Carbohydrate Resource Bank (MCRB), Konkuk University, 120 Neungdong-ro, Gwangjin-gu, Seoul 05029, Republic of Korea; qkrtkddlf503@naver.com (S.-I.P.); rudgh971225@naver.com (K.K.); rsm127@naver.com (S.R.); 2 Department of System Biotechnology, Konkuk University, 120 Neungdong-ro, Gwangjin-gu, Seoul 05029, Republic of Korea

**Keywords:** succinoglycan, tannic acid grafting, antioxidant and antibacterial activity, self-healing hydrogel, drug delivery

## Abstract

Tannic acid grafting provides a practical strategy for introducing bioactive phenolic functionality into microbial polysaccharides for multifunctional hydrogel design. Herein, tannic acid-modified succinoglycan (SG-TA) was prepared through an ascorbic acid/ H_2_O_2_-mediated free-radical process. Spectroscopic, thermal, and purification-control analyses were consistent with covalent incorporation of tannic acid-derived moieties into SG, while characteristic structural features of the SG framework remained evident after modification. SG-TA exhibited tannic acid-equivalent phenolic contents of up to 321.9 mg TAE/g and markedly enhanced antioxidant and antibacterial activities compared with native SG. SG-TA was subsequently incorporated into a poly(vinyl alcohol) (PVA)/borax network to form dynamic SG-TA/PVA/borax (STPB) hydrogels based on reversible interactions. The hydrogels exhibited composition-dependent viscoelasticity, rapid rheological recovery, macroscopic self-rejoining, enhanced deformability, antioxidant and antibacterial functionality, and preliminary cytocompatibility. Time-dependent phenolic release showed that SG-TA-derived phenolic species were partially released from the network, indicating contributions from both matrix-associated and releasable functionality. The reversible network also enabled pH- and glucose-responsive release of 5-fluorouracil as a model small-molecule drug. These findings demonstrate the potential of SG-TA as an intrinsically bioactive microbial polysaccharide for multifunctional, self-healing, and stimuli-responsive drug-delivery hydrogels.

## 1. Introduction

Stimuli-responsive hydrogels have attracted considerable interest for controlled drug delivery due to their ability to regulate release behavior in response to pH, glucose, and oxidative stress [1,2]. Oxidative stress and microbial contamination are also important challenges in many biomedical settings, motivating the development of hydrogel systems that combine responsive delivery with antioxidant and antibacterial functionality [3,4,5,6]. Exopolysaccharides are attractive candidates for such systems because of their biocompatibility, structural diversity, water-retention capacity, and chemical modifiability. Recent studies have expanded their use from conventional rheological modifiers to functional components of responsive hydrogels and drug-delivery systems [7,8]. However, many exopolysaccharide-based hydrogels still rely on the physical incorporation of external bioactive agents to achieve antioxidant or antibacterial functions, which may limit long-term stability and complicate structure–function control [9,10,11]. Therefore, engineering bioactivity directly into the polysaccharide backbone represents a promising strategy for developing intrinsically functional hydrogel platforms.

Succinoglycan (SG) is an anionic microbial exopolysaccharide produced by bacteria including *Sinorhizobium*, *Rhizobium*, and *Agrobacterium*. Its octasaccharide repeating unit is composed mainly of glucose and galactose and contains native succinyl, acetyl, and pyruvyl substituents that influence its physicochemical properties and chemical reactivity [12,13,14,15]. Beyond its physicochemical advantages, SG-based materials have also been associated with biological effects, including anti-inflammatory activity in skin-injury models and potential antitumor or immunomodulatory effects reported for succinoglycan riclin [16,17,18,19]. Nevertheless, further functionalization is desirable for applications requiring stronger antioxidant and antibacterial performance. Chemical modification of SG therefore offers an opportunity to expand its role from a primarily structural microbial polysaccharide to a multifunctional biomaterial component.

To enhance the bioactivity of polysaccharide-based systems, polyphenolic compounds have been widely utilized due to their antioxidant and antibacterial properties and their ability to form multiple intermolecular interactions through abundant hydroxyl groups [20,21]. Tannic acid (TA), which contains abundant galloyl and phenolic hydroxyl groups, has been extensively employed in biomaterials for antioxidant, antibacterial, and crosslinking functions [22]. Although chemical conjugation of polyphenols to polysaccharides has been investigated in several systems, much of the reported work has focused on conventional polysaccharides such as chitosan [23,24]. In comparison, radical-mediated incorporation of TA into structurally distinct microbial exopolysaccharides such as SG remains comparatively unexplored. Free-radical-mediated grafting provides a practical route for introducing phenolic functionality into polysaccharides [25,26]. In H_2_O_2_/ascorbic acid redox systems, reactive species can generate active sites along polysaccharide chains and facilitate coupling with phenolic compounds under relatively mild conditions [27,28]. Such modification therefore provides a potential strategy for integrating TA-derived functionality directly into SG while enabling investigation of relationships among chemical modification, polymer properties, and biological activity [29].

In this study, tannic acid-modified succinoglycan (SG-TA) was prepared through free-radical-mediated modification and characterized in terms of its structural, physicochemical, antioxidant, and antibacterial properties. SG-TA was subsequently incorporated into a poly(vinyl alcohol) (PVA)/borax system to form dynamic hydrogels based on reversible borate–diol and secondary intermolecular interactions [30,31]. We hypothesized that radical-mediated incorporation of tannic acid-derived moieties into SG would impart intrinsic antioxidant and antibacterial functionality and enable SG-TA to participate in a dynamic PVA/borax network capable of self-healing and pH- and glucose-responsive drug release. The resulting hydrogels were evaluated for viscoelastic behavior, deformability, self-healing, swelling, bioactivity, cytocompatibility, and pH- and glucose-responsive release of 5-fluorouracil. Through this approach, the present study aimed to establish SG-TA as an intrinsically bioactive microbial polysaccharide component for multifunctional and stimuli-responsive hydrogel systems.

## 2. Results and Discussion

### 2.1. Characterization of SG-TA

Before tannic acid grafting, the purified SG backbone was evaluated with reference to the known structure of succinoglycan. As shown in Figure S1a, the purified SG showed low UV absorbance without distinct peaks at 260 or 280 nm, suggesting low levels of nucleic acid- and protein-related impurities [32,33]. The FTIR spectrum of SG showed typical polysaccharide absorption bands, including broad O–H stretching at around 3300 cm^−1^, C–H stretching near 2900 cm^−1^, C=O stretching at approximately 1732 cm^−1^, COO^-^ stretching bands at 1622 and 1378 cm^−1^, and a strong C–O–C vibration near 1040 cm^−1^ (Figure S1b). As shown in Figure S1c, SG exhibited characteristic polysaccharide backbone signals at 3.0–5.5 ppm in the ^1^H NMR spectrum, together with substituent-related signals assigned to succinyl, acetyl, and pyruvyl groups, consistent with previous reports [34,35]. The apparent molecular weight (M_w_) of the purified SG, determined by GPC using pullulan standards, was approximately 324.3 kDa (Table 1). These results support that the purified SG retained the characteristic structural features of succinoglycan and was suitable for subsequent tannic acid grafting.

**Figure 1:**
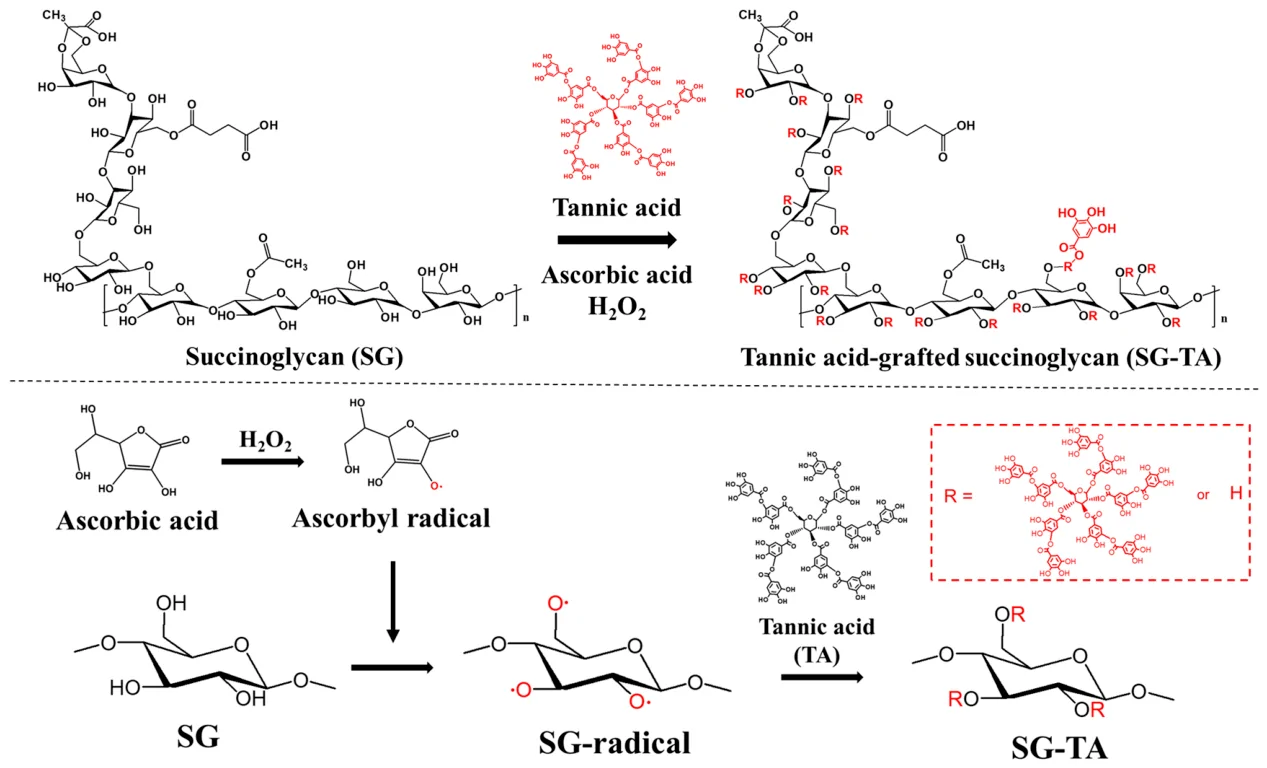
Proposed radical-mediated modification pathways for tannic acid incorporation into succinoglycan using the ascorbic acid/H_2_O_2_ redox system.

Tannic acid-grafted succinoglycan (SG-TA) was prepared by free-radical grafting using an ascorbic acid/H_2_O_2_ redox system, as illustrated in Figure 1. In this system, ascorbic acid reacts with H_2_O_2_ to generate ascorbyl radicals, which subsequently induce radical formation on the SG backbone [36]. The generated SG macroradicals are proposed to serve as reactive sites for radical-mediated coupling with TA-derived species [37]. Because the exact SG–TA linkage and reaction pathway were not directly identified, Figure 1 is presented as a proposed mechanism that includes possible radical coupling as well as competing oxidation, TA self-reaction, and non-covalent interactions. This radical-mediated modification provides a practical route for introducing phenolic functionality into SG without additional coupling agents.

**Figure 2:**
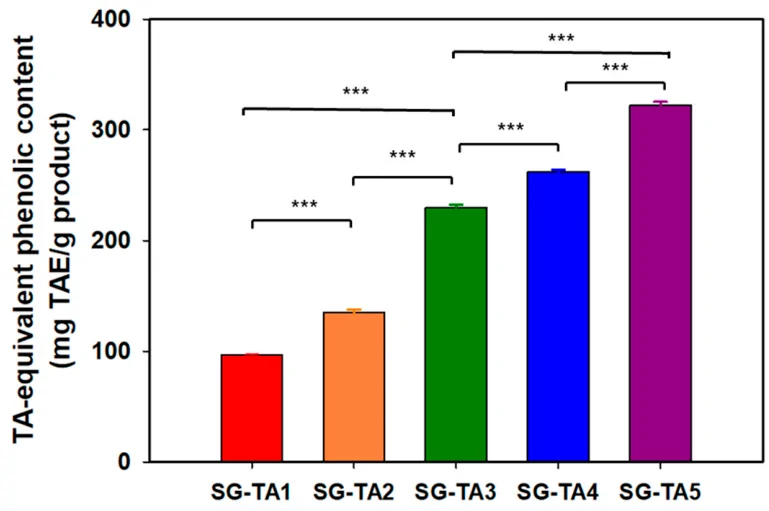
Tannic acid-equivalent (TAE) phenolic content of SG-TA samples. Data are presented as mean ± SD (*n* = 3). Statistical significance was evaluated by one-way ANOVA followed by Tukey’s multiple-comparison test. *** *p* < 0.001.

The TA-equivalent (TAE) phenolic content of SG-TA samples was quantified using the Folin–Ciocalteu assay (Figure 2). The TAE values increased progressively from SG-TA1 to SG-TA5, indicating higher phenolic content in the purified SG-TA products with increasing TA feed. The detailed TAE values are summarized in Table 1. The TAE phenolic content increased from 96.50 ± 0.49 mg TAE/g product for SG-TA1 to 321.97 ± 3.40 mg TAE/g product for SG-TA5 (Figure 2 and Table 1). The increase was statistically significant among the SG-TA formulations (*p* < 0.001). However, the TAE response did not increase proportionally with the initial TA feed. To provide a clearer synthesis mass balance, Table 1 additionally summarizes the recovered product yield, TAE-based apparent grafting efficiency, and estimated unretained TA fraction. Because these quantities are derived from the Folin–Ciocalteu response, they are interpreted as apparent, TAE-based estimates rather than direct measurements of covalently grafted TA.

This non-linear behavior may be attributed to the limited number of radical-accessible sites on the SG backbone and the steric hindrance imposed by bulky tannic acid molecules at higher feed ratios [38,39]. In contrast to the progressive increase in TAE values, the apparent molecular weights of the SG-TA samples remained within a relatively narrow range of 336.2–379.0 kDa, compared with 324.3 kDa for native SG. The corresponding GPC chromatograms and molecular-weight distribution parameters are presented in Figure S2, with PDI values ranging from approximately 1.36 to 1.46 across native SG and the SG-TA derivatives. Notably, none of the SG-TA samples exhibited an apparent molecular weight lower than that of native SG. In H_2_O_2_/ascorbic acid redox systems, phenolic coupling and radical-induced chain scission may occur concurrently, and the resulting molecular-weight response can vary depending on the reaction conditions, with either increases or decreases in apparent molecular weight reported according to the relative contributions of grafting and chain degradation [40]. Moreover, conventional GPC calibrated with pullulan standards provides an apparent molecular weight based on relative hydrodynamic behavior rather than an absolute molecular mass. Thus, changes in chain conformation, hydrodynamic volume, or intermolecular association following TA incorporation may influence the measured values [41,42]. The non-proportional relationship between TA feed and apparent molecular weight therefore likely reflects the combined effects of TA-derived modification, possible limited radical-induced chain changes, and altered hydrodynamic behavior rather than a simple increase in polymer mass. Overall, the molecular-weight results indicate that SG-TA retained a macromolecular size range comparable to that of native SG, although limited radical-induced changes in chain architecture may have occurred during modification.

**Figure 3:**
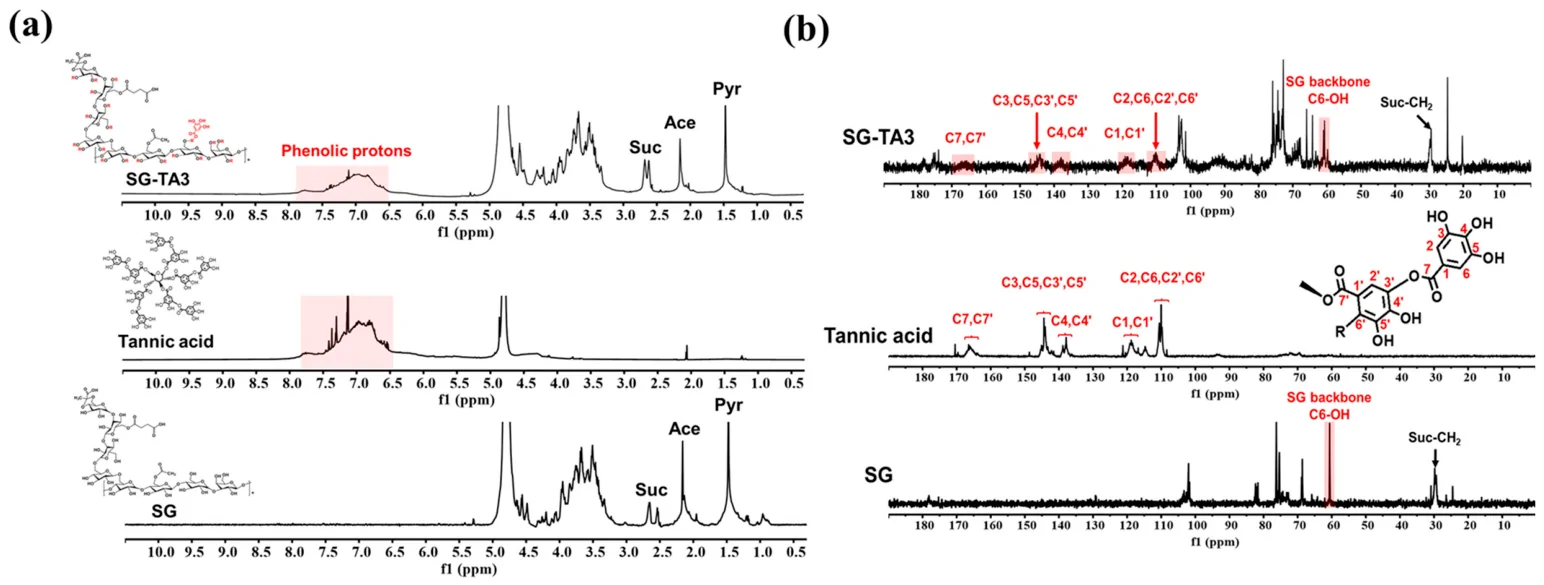
Structural characterization of SG, tannic acid, and SG-TA3 using (**a**) ^1^H and (**b**) ^13^C NMR spectra.

The NMR spectra of SG, tannic acid, and SG-TA were recorded to confirm the chemical structure (Figure 3). As shown in Figure 3a, the ^1^H NMR spectrum of SG exhibited characteristic signals of the polysaccharide backbone in the region of 3.0–5.5 ppm, corresponding to protons of the sugar ring units and glycosidic linkages. Signals at approximately 2.7 ppm, 2.1 ppm, and 1.5 ppm were respectively assigned to the methylene protons of succinyl groups, the methyl protons of acetyl substituents, and the methyl protons of pyruvyl groups [34,35]. The ^1^H NMR spectra of SG-TA samples showed new resonances in the aromatic region of approximately 6.5–7.5 ppm. These peaks correspond to the phenolic protons of tannic acid [38,43]. In Figure S3, the intensity of the aromatic signals increased with increasing TA feed ratio, suggesting a progressive incorporation of tannic acid moieties into the succinoglycan matrix. The ^1^H NMR spectra of a physical mixture of SG and tannic acid were compared before and after dialysis in Figure S4. The aromatic signals observed before dialysis disappeared after dialysis, indicating that physically mixed tannic acid was effectively removed. Together with the reaction-induced NMR changes and the established radical-mediated grafting chemistry, the persistence of these signals after dialysis is consistent with stable covalent incorporation of TA-derived moieties rather than simple physical mixing [44]. To further support the structural modification, ^13^C NMR spectra of SG, SG-TA3, and tannic acid were compared in Figure 3b. SG-TA3 retained the major polysaccharide carbon signals of SG, while additional resonances appeared in the aromatic carbon region corresponding to tannic acid [45,46]. The reduced intensity of the C6-related signal near 60 ppm after tannic acid grafting indicates that the local C6-associated hydroxymethyl environment was affected by the reaction and suggests that this region may participate in the modification process [47,48,49]. Together, these spectral features support the stable incorporation of TA-derived moieties into SG while preserving the major characteristic spectral features of the polysaccharide.

**Figure 4:**
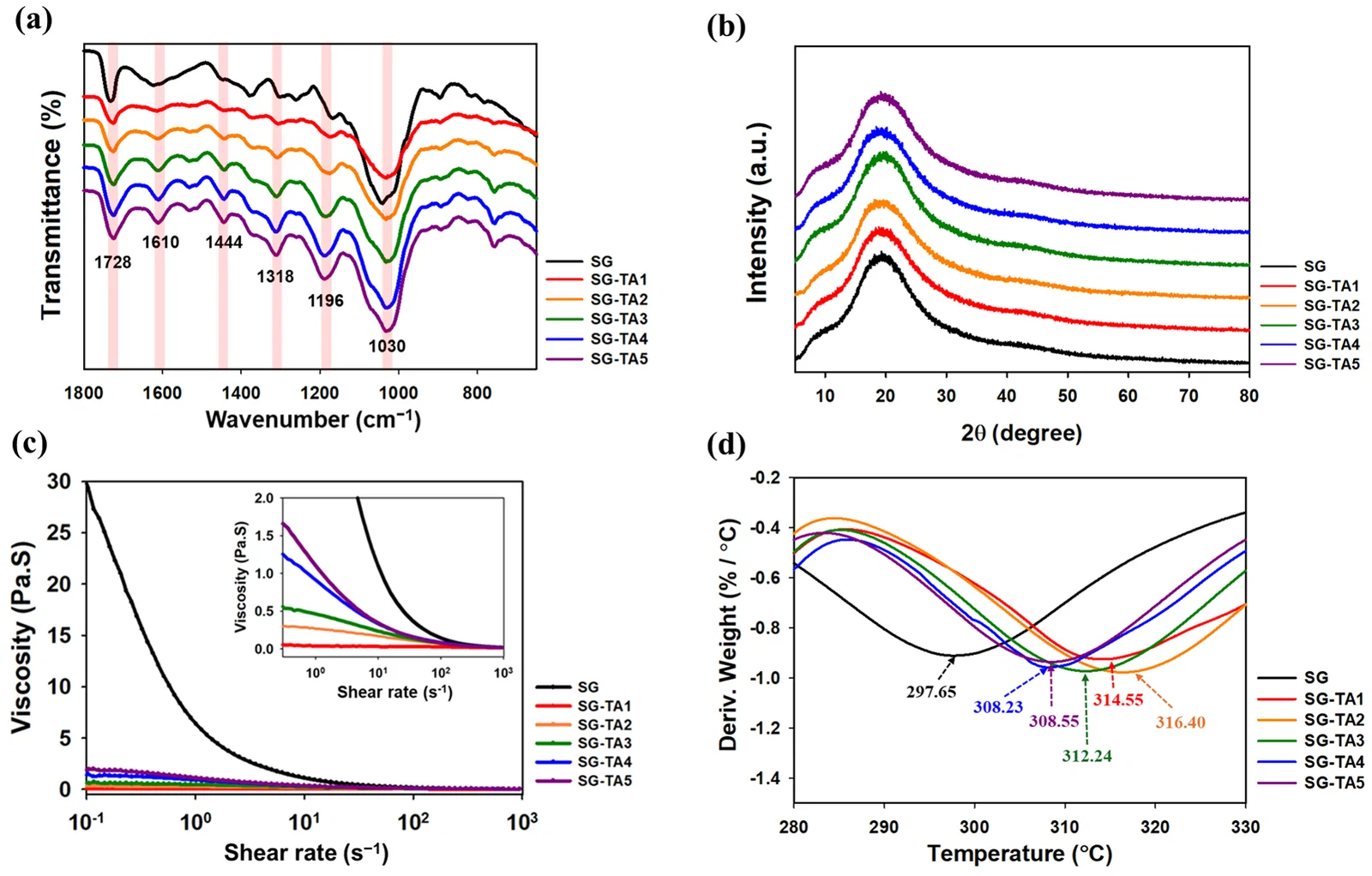
Characterization of SG and SG-TA samples: (**a**) FTIR spectra, (**b**) XRD patterns, (**c**) shear viscosity of SG and SG-TA solutions as a function of shear rate (0.1–1000 s^−1^) at 25 °C, and (**d**) DTG curves.

Fourier transform infrared (FTIR) spectra of SG and SG-TA samples were analyzed in Figure 4a. Native SG showed typical polysaccharide-related absorption bands, including broad O–H stretching around 3400 cm^−1^, C–O–C stretching vibrations near 1030 cm^−1^ and a carbonyl stretching band near 1720 cm^−1^ arising from the succinyl groups. In SG-TA samples, the carbonyl band slightly shifted to approximately 1728 cm^−1^ with increased intensity. Additional bands appeared at 1610 and 1444 cm^−1^, corresponding to aromatic C=C stretching vibrations. Bands at 1318 and 1196 cm^−1^ were attributed to phenolic C–O vibrations [37,50,51]. The intensities of these bands increased with increasing TA feed ratio, supporting the presence of increasing amounts of TA-derived phenolic functionality in the purified SG-TA samples. These FTIR results are consistent with the TAE and NMR analyses and provide complementary evidence for chemical modification of SG that is consistent with the proposed grafting process.

In Figure 4b, X-ray diffraction (XRD) patterns of SG and SG-TA samples were analyzed to evaluate changes in structural order. All samples exhibited broad diffraction halos centered at 2θ ≈ 18–22°, indicating that both SG and SG-TA possess predominantly amorphous structures. No distinct crystalline peaks were observed after tannic acid grafting, suggesting that the overall backbone structure of SG remained largely unchanged. A broadening of the diffraction halo and slight variations in peak intensity were observed with increasing tannic acid content. These changes suggest that tannic acid grafting affected intermolecular chain packing and hydrogen-bonding interactions without inducing crystallization or major structural rearrangement. The H_2_O_2_/ascorbic acid system generates reactive radical species that can modify intermolecular interactions and local chain organization within the polysaccharide matrix, resulting in reduced structural ordering and a more amorphous structure [40,52].

### 2.2. Rheological Properties of SG-TA

The rheological properties of SG and SG-TA solutions were evaluated through shear viscosity and amplitude sweep measurements. As shown in Figure 4c, SG exhibited higher viscosity across the entire shear rate range (0.1–1000 s^−1^), along with pronounced shear-thinning behavior. In contrast, all SG-TA samples displayed lower viscosity. Because the SG-TA samples were exposed to both the H_2_O_2_/ascorbic acid radical treatment and TA during modification, the reduction in viscosity cannot be attributed exclusively to TA incorporation. Rather, it may reflect combined changes in chain conformation, intermolecular association, and possible radical-induced structural changes during the modification process [53,54]. Interestingly, as the TA-equivalent phenolic content increased from SG-TA1 to SG-TA5, the viscosity gradually increased, although it remained lower than that of SG. This composition-dependent increase may be associated with the increasing phenolic functionality and accompanying changes in intermolecular association, including possible hydrogen-bonding interactions [46,55]. The amplitude sweep results further support this behavior (Figure S5). All samples exhibited typical strain-dependent viscoelastic responses, with SG showing the highest storage modulus (G′), consistent with stronger intermolecular association in the native SG solution. In contrast, SG-TA samples showed reduced G′ values, consistent with the decreased viscosity observed in shear measurements. However, with increasing TA-equivalent phenolic content, both G′ and G″ values gradually increased, indicating a composition-dependent increase in viscoelastic response among the SG-TA derivatives. The progressive increase in viscosity and oscillatory moduli across the SG-TA series may reflect composition-dependent changes in intermolecular associations associated with increasing phenolic functionality. To further assess the contribution of the redox treatment itself, SG subjected to the H_2_O_2_/ascorbic acid treatment in the absence of TA (SG-redox) was additionally examined. SG-redox exhibited markedly reduced viscosity relative to untreated SG (Figure S6), indicating that the redox treatment itself substantially contributed to the decrease in viscosity [56]. Accordingly, the lower viscosity of SG-TA relative to native SG likely reflects the combined effects of redox-induced changes in the SG chains and TA-derived intermolecular interactions, whereas the progressive increase across the SG-TA series may be associated with increasing phenolic functionality.

### 2.3. Thermal Stability of SG-TA

The thermal degradation behavior of SG and SG-TA was examined using thermogravimetric analysis (TGA) and derivative thermogravimetric (DTG) curves. All samples exhibited a similar multi-step weight loss pattern, consisting of an initial weight loss below 100 °C followed by a major weight loss in the range of approximately 250–330 °C (Figure S7). The initial weight loss is attributed to the removal of physically adsorbed and bound water, whereas the major weight loss corresponds to the decomposition of the polysaccharide structure. In Figure 4d, the maximum degradation temperature (T_max_) increased from 297.65 °C for SG to a maximum of 316.40 °C for SG-TA2, showing that the thermal degradation behavior was altered after radical-mediated TA modification. The increased T_max_ observed for the lower- to intermediate-TA formulations may be associated with the contribution of thermally stable aromatic TA-derived structures and composition-dependent intermolecular interactions, including hydrogen bonding [37,40,57]. At higher TA-equivalent phenolic contents, T_max_ decreased slightly from the maximum observed for SG-TA2. Rather than indicating a simple monotonic relationship between TA content and thermal stability, this trend suggests that thermal behavior reflects the combined effects of TA-derived functionality, changes in intermolecular organization, and possible radical-induced structural changes during modification. Accordingly, the DTG results are interpreted as complementary evidence that the chemical and thermal environment of SG was modified.

### 2.4. Antioxidant Activities of SG-TA

**Figure 5:**
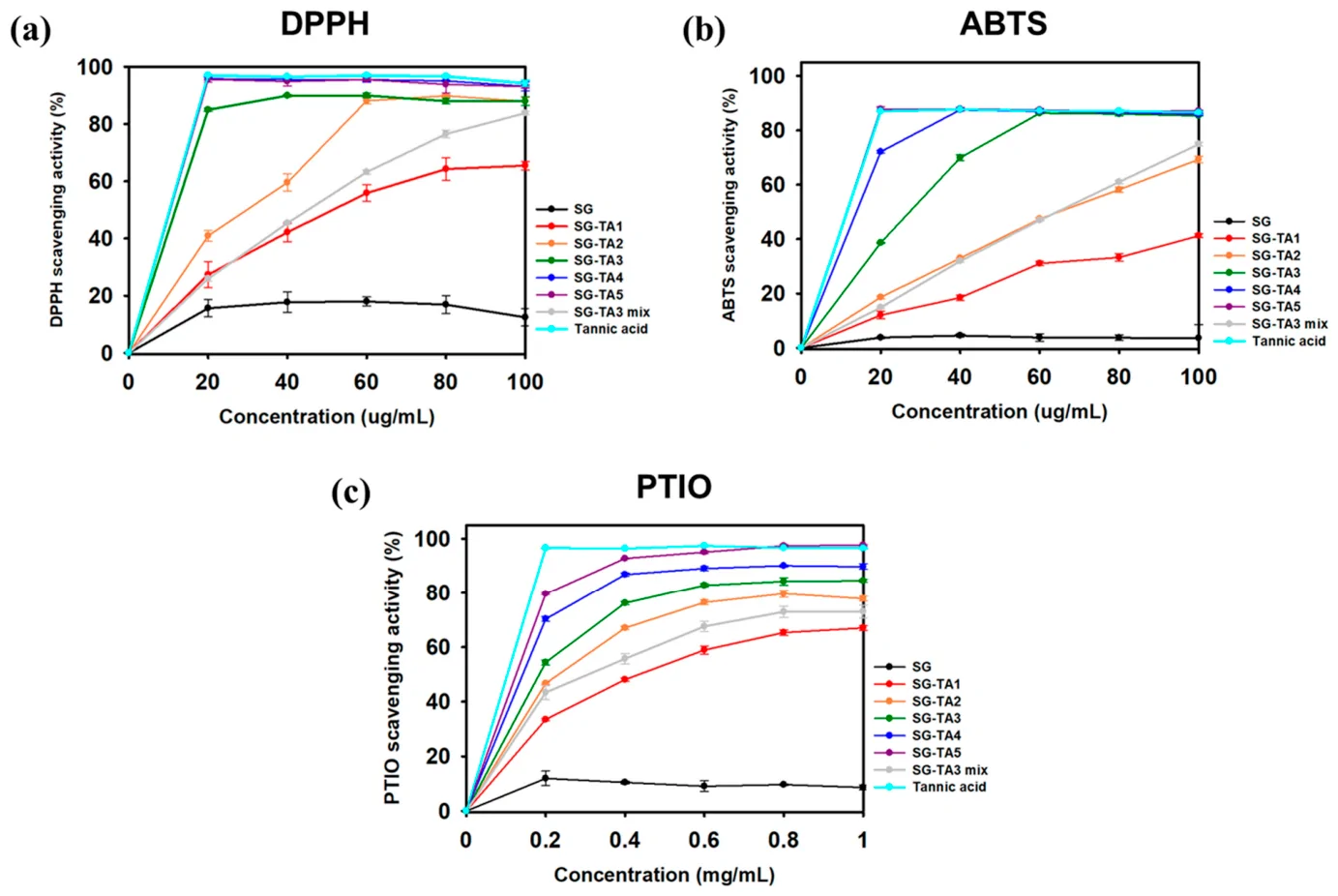
Antioxidant activities of SG and SG-TA samples evaluated by (**a**) DPPH, (**b**) ABTS, and (**c**) PTIO assays. The SG-TA3 physical mixture and free tannic acid were included as comparison controls. Data are presented as mean ± SD (*n* = 3).

The antioxidant activities of SG and SG-TA derivatives were evaluated using DPPH, ABTS, and PTIO radical-scavenging assays (Figure 5). Native SG showed relatively low scavenging activity, whereas the SG-TA derivatives exhibited higher activity across the tested concentration ranges. In the DPPH assay at 20 μg/mL, scavenging activity increased from 27.3% for SG-TA1 to 84.9% for SG-TA3 and approximately 95% for SG-TA4 and SG-TA5 (Figure 5a). Similarly, in the ABTS assay at the same concentration, the activity increased from 12.0% for SG-TA1 to 38.7% for SG-TA3 and 87.8% for SG-TA5 (Figure 5b). At higher sample concentrations, particularly for the higher-TA formulations, the DPPH and ABTS responses approached a plateau, resulting in smaller differences among the formulations. The overall increase in antioxidant activity with TA-equivalent phenolic content is consistent with the contribution of TA-derived phenolic groups to radical scavenging [38,58]. To distinguish the antioxidant response of radical-mediated SG-TA modification from that of simple SG/TA mixing, an SG-TA3 physical-mixture control was prepared using the same SG/TA composition as SG-TA3 but without the H_2_O_2_/ascorbic acid redox initiator. The physical-mixture control showed consistently lower radical-scavenging activity than SG-TA3. At 20 μg/mL, DPPH scavenging was 84.9% for SG-TA3 compared with 25.9% for the physical mixture, while the corresponding ABTS values were 38.7% and 14.9%, respectively. Thus, simple physical mixing of SG and TA in the absence of radical-mediated modification did not reproduce the antioxidant behavior observed for SG-TA3 under the tested conditions.

The PTIO assay was additionally employed as a complementary radical-scavenging method because PTIO is a stable nitronyl nitroxide radical with reaction characteristics distinct from those of DPPH and ABTS [59,60]. At 0.2 mg/mL, scavenging activity was 33.5%, 54.4%, and 79.6% for SG-TA1, SG-TA3, and SG-TA5, respectively, compared with 11.9% for native SG. SG-TA3 also showed higher PTIO scavenging than the corresponding physical-mixture control, with values of 54.4% and 43.4%, respectively. The wider separation among the SG-TA formulations in the PTIO assay further demonstrated the dependence of antioxidant behavior on SG-TA composition.

### 2.5. Antibacterial Activity of SG-TA

**Figure 6:**
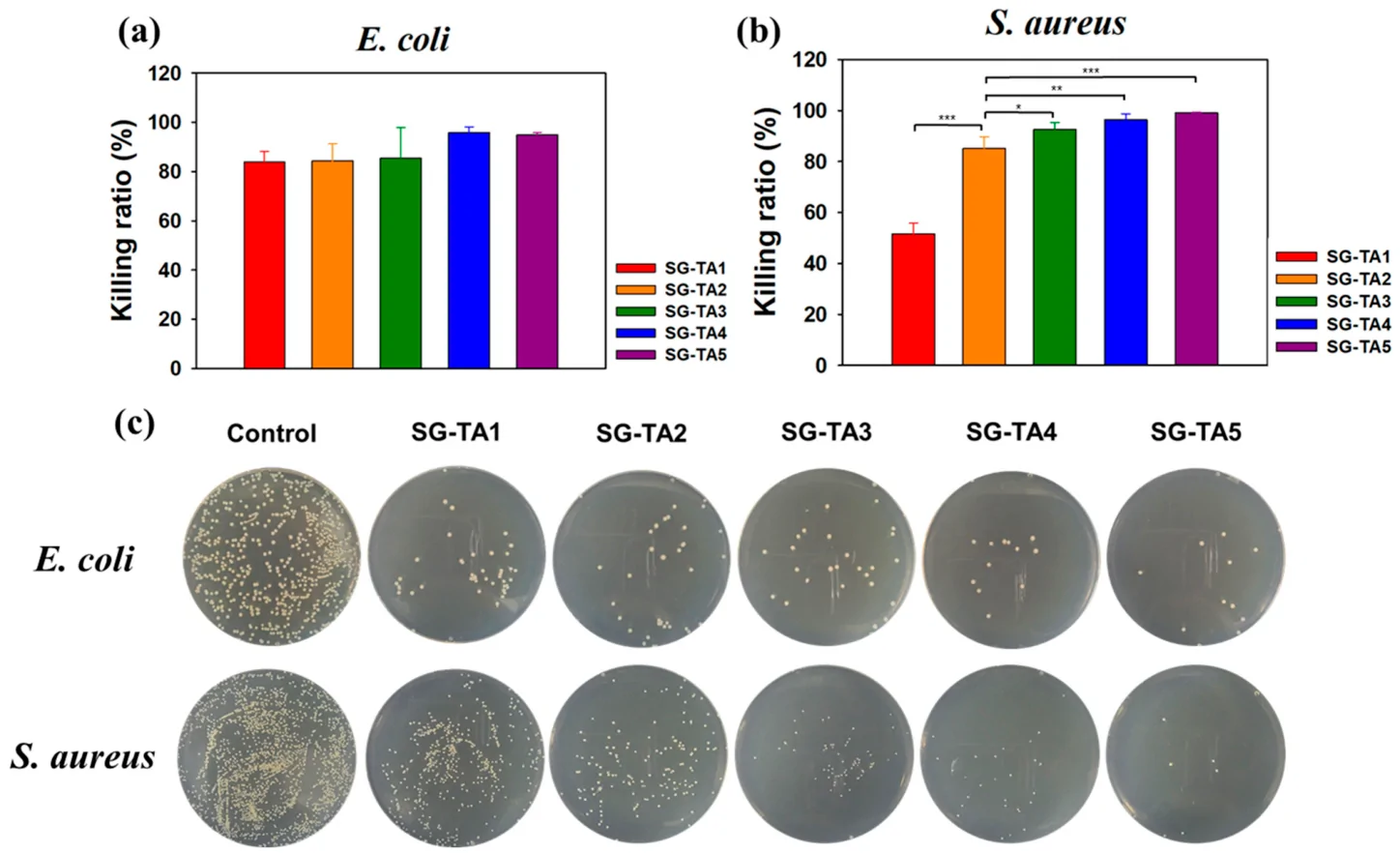
Antibacterial activity of SG-TA derivatives against (**a**) *E. coli* and (**b**) *S. aureus*, together with (**c**) representative CFU images. Data are presented as mean ± SD (*n* = 3). Statistical significance was evaluated by one-way ANOVA followed by Tukey’s multiple-comparison test where applicable (* *p* < 0.05, ** *p* < 0.01, and *** *p* < 0.001).

In Figure 6, the antibacterial activity of SG-TA samples was quantitatively evaluated by colony-forming unit (CFU) counting against *E. coli* and *S. aureus*. For *E. coli*, the killing ratios were 83.98%, 84.25%, 85.47%, 95.79%, and 94.81% for SG-TA1–SG-TA5, respectively. Because substantial activity was already observed for SG-TA1, further increases in TA-equivalent phenolic content produced only modest changes, with no significant differences among the SG-TA formulations (*p* > 0.05). In contrast, *S. aureus* showed a more distinct composition-dependent response, with killing ratios of 51.73%, 85.17%, 93.50%, 96.39%, and 99.00% for SG-TA1–SG-TA5, respectively. These results indicate substantial antibacterial activity of SG-TA against both bacterial strains, with a more pronounced composition-dependent response observed for *S. aureus*. The antibacterial activity is consistent with the contribution of TA-derived phenolic functionality, which may act through membrane perturbation, protein interactions, metal ion chelation, and related mechanisms reported for tannic acid-containing systems [61,62]. Overall, radical-mediated TA modification provided SG with substantial antibacterial functionality against both Gram-negative and Gram-positive bacteria.

### 2.6. Formation and Macroscopic Stability of STPB Hydrogels

**Figure 7:**
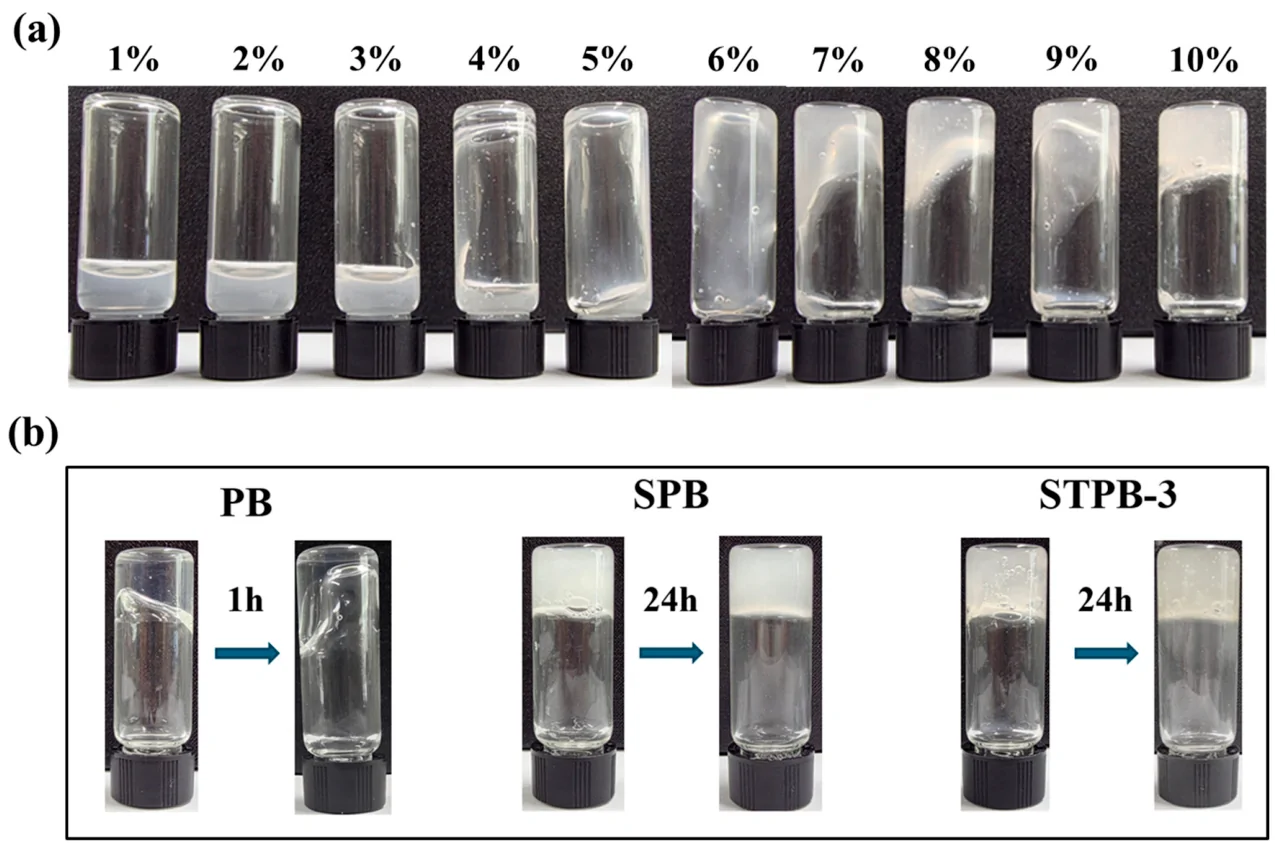
Macroscopic evaluation of hydrogel formation and stability. (**a**) Vial-inversion test of formulations prepared with PVA concentrations ranging from 1 to 10% (*w*/*v*) under otherwise identical formulation conditions. (**b**) Representative photographs showing the macroscopic structural stability of PB, SPB, and STPB-3 after standing at room temperature. PB showed substantial structural relaxation within 1 h, whereas SPB and STPB-3 retained their macroscopic form for at least 24 h.

Hydrogel formation was first evaluated macroscopically using the vial-inversion method at PVA concentrations ranging from 1 to 10% (*w*/*v*) under otherwise identical formulation conditions (Figure 7a). The resistance to flow progressively increased with increasing PVA concentration, whereas formulations below 10% did not maintain a stable self-supporting state upon inversion. At a 10% (*w*/*v*) PVA precursor concentration, corresponding to a final PVA concentration of approximately 7.69% (*w*/*v*) after borax addition, the formulation maintained its position without observable flow and was therefore identified as the minimum precursor PVA concentration producing a self-supporting gel under the tested formulation conditions [63].

The macroscopic stability of the selected formulations was further compared over time (Figure 7b). PB underwent substantial structural relaxation within approximately 1 h, whereas SPB and STPB-3 retained their macroscopic form for at least 24 h. This observation suggests that incorporation of SG or SG-TA improves the short-term structural stability of the PVA/borax-based network under the tested conditions.

### 2.7. Rheological and Physicochemical Properties of STPB Hydrogels

**Figure 8:**
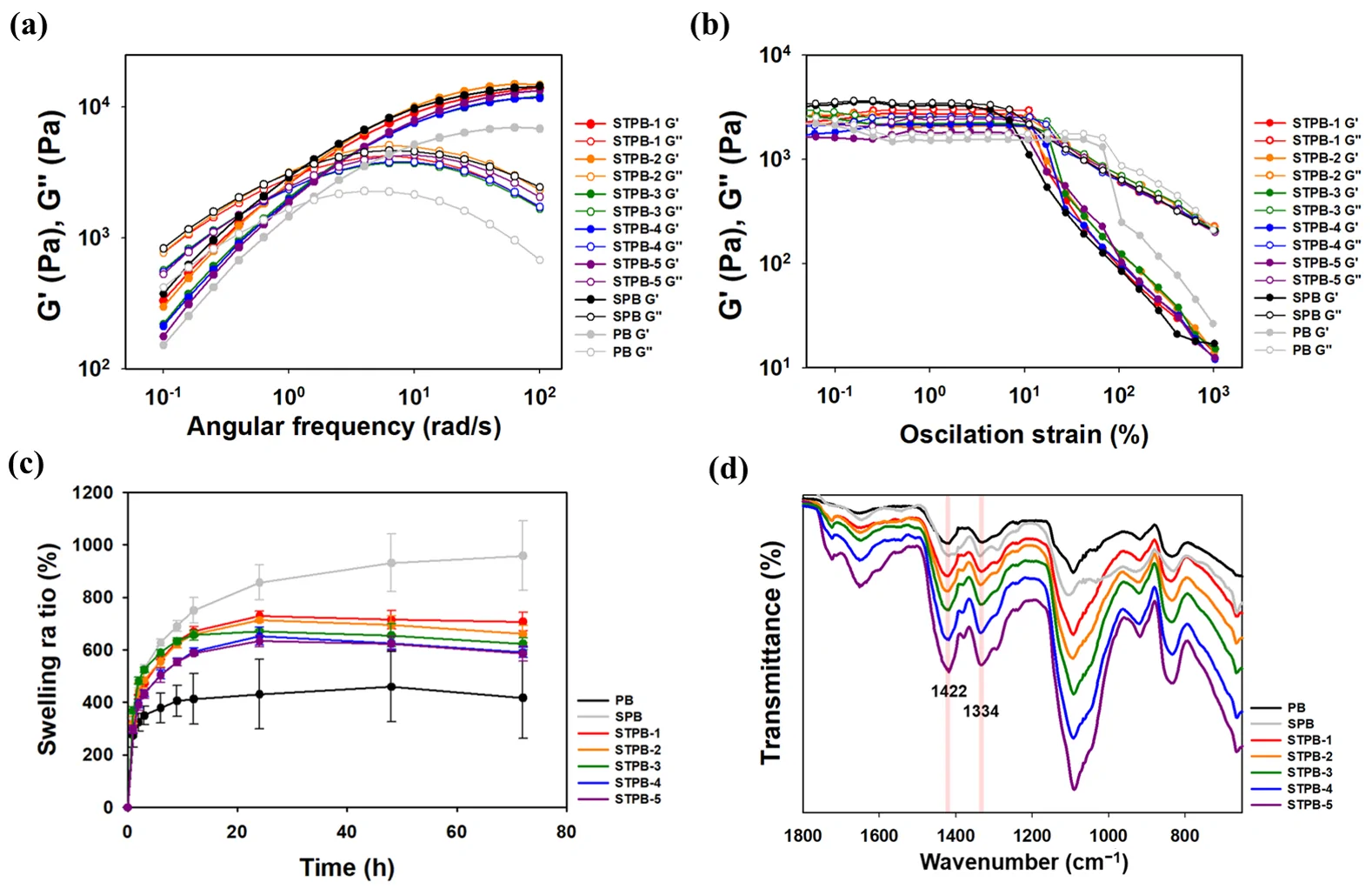
Physicochemical and rheological characterization of PB, SPB, and STPB hydrogels. (**a**) Frequency-dependent storage (G′) and loss (G″) moduli, (**b**) strain-dependent viscoelastic behavior obtained from amplitude-sweep measurements, (**c**) swelling ratios measured over 72 h, and (**d**) FTIR spectra of PB, SPB, and STPB-1–STPB-5. Data in (**c**) are presented as mean ± SD (*n* = 3 independent samples).

The viscoelastic behavior of PB, SPB, and STPB hydrogels was evaluated by frequency- and amplitude-sweep measurements (Figure 8a,b). In the frequency sweep, all formulations exhibited a frequency-dependent transition in viscoelastic behavior (Figure 8a). At low angular frequencies, G″ exceeded G′, indicating predominantly viscous behavior over longer deformation time scales. With increasing angular frequency, G′ progressively increased relative to G″, and a G′/G″ crossover was observed at approximately 1–2 rad/s, with small composition-dependent differences among the formulations. Above the crossover region, G′ became dominant over G″. This frequency-dependent transition was further reflected in the loss tangent (tan δ), which decreased from values above 1 at low frequencies to below 1 at higher frequencies, consistent with the transition from viscous-dominant to elastic-dominant behavior (Figure S8). This behavior is consistent with a transient dynamically crosslinked network in which reversible interactions can relax at low frequencies but increasingly contribute to elastic behavior at shorter experimental time scales [63,64]. The magnitude of the viscoelastic moduli also depended on hydrogel composition. At 1 rad/s, the G′ values of PB, SPB, STPB-1, STPB-2, STPB-3, STPB-4, and STPB-5 were 1.46, 2.91, 2.60, 2.63, 2.02, 1.95, and 1.88 kPa, respectively, while the corresponding G″ values were 1.66, 3.11, 2.86, 3.15, 2.36, 2.35, and 2.44 kPa. Increasing TA-equivalent phenolic content of the incorporated SG-TA derivatives across the STPB series was accompanied by a general decrease in G′ and G″, particularly in the higher-TA formulations. This trend suggests that incorporation of increasing amounts of TA-derived phenolic functionality alters the effective dynamic-network interactions. Participation of phenolic hydroxyl groups in borate-mediated interactions and changes in hydrogen bonding or polymer-chain organization may contribute to this behavior; however, competitive coordination of TA-derived groups with PVA diols was not directly demonstrated in the present study [65,66,67]. Accordingly, the decrease in G′ and G″ at higher phenolic contents is more appropriately interpreted as a modification of effective dynamic-network connectivity rather than simply as an increase or decrease in overall crosslinking density.

Time-sweep measurements of freshly prepared hydrogels further revealed composition-dependent network development (Figure S9). PB and SPB exhibited G′ values above G″ throughout the measured period, with gradual increases in the moduli. In contrast, STPB-3 initially showed G″ > G′, followed by a rapid G′/G″ crossover at approximately 35–40 s and a marked increase in G′ thereafter, indicating rapid establishment of an elastic-dominant network following the initial structural rearrangement [68].

The amplitude-sweep measurements further demonstrated composition-dependent strain responses (Figure 8b). All formulations maintained relatively stable moduli within the low-strain region, followed by pronounced decreases in G′ and G″ at larger strains as the dynamic network was disrupted. At approximately 1% strain, the G′ values of PB, SPB, STPB-1, STPB-2, STPB-3, STPB-4, and STPB-5 were 1.51, 3.30, 2.72, 2.08, 2.20, 2.12, and 1.77 kPa, respectively, while the corresponding G″ values were 1.71, 3.46, 2.99, 2.54, 2.54, 2.55, and 2.37 kPa. The differences in the onset and magnitude of modulus decay among PB, SPB, and the STPB formulations indicate that incorporation of SG and SG-TA modifies the deformation behavior of the PVA/borax network. The altered strain tolerance of the STPB formulations may arise from the introduction of additional reversible interactions associated with SG-TA, which can reorganize or dissipate stress under deformation rather than behaving as permanent junctions. This interpretation is consistent with the dynamic borate-mediated and hydrogen-bonding interactions expected within the STPB network [65,66].

The hydration behavior of the hydrogels was further evaluated by swelling measurements over 72 h (Figure 8c). PB exhibited the lowest swelling, whereas incorporation of native SG markedly increased water uptake in SPB. The STPB formulations also showed substantial swelling but exhibited lower equilibrium swelling ratios than SPB. Within the STPB series, the swelling ratio generally decreased with increasing TA content, with STPB-1 showing the highest and STPB-5 the lowest swelling among the SG-TA-containing formulations. The enhanced swelling of SPB is consistent with the high hydrophilicity of SG, whereas the lower swelling of the STPB formulations suggests that TA-derived phenolic functionality alters the polymer–water interaction environment and intermolecular associations within the network. Multiple hydrogen-bonding and borate-mediated interactions involving TA-derived phenolic groups may favor intermolecular association and reduce the effective availability of hydrophilic groups for water uptake.

FTIR spectroscopy was additionally used to examine composition-dependent spectral changes in the hydrogel formulations (Figure 8d). PB, SPB, and the STPB hydrogels exhibited broadly similar spectral profiles characteristic of the PVA/borax-based network, while systematic changes were observed in the regions around 1422 and 1334 cm^−1^ after incorporation of SG and SG-TA. These spectral variations are consistent with changes in the local hydroxyl- and borate-associated interaction environment within the hydrogel network. The progressively altered band profiles across the STPB series further indicate that SG-TA incorporation modifies intermolecular interactions within the PVA/borax matrix. Together with the rheological and swelling results, these FTIR changes suggest that SG-TA incorporation alters the borate-mediated and hydrogen-bonding interaction environment within the hydrogel network.

Based on these composition-dependent rheological characteristics together with the subsequent bioactivity results, STPB-3 was selected as the representative formulation for self-healing and drug-release studies. At 1 rad/s, STPB-3 retained higher G′ than STPB-4 and STPB-5, while the antioxidant activities of the STPB formulations were already near plateau levels across the series. Therefore, STPB-3 was selected as an intermediate formulation that retained substantial bioactivity while avoiding the greater reduction in viscoelastic moduli observed in the higher-TA formulations.

### 2.8. Morphological Characterization of STPB Hydrogels

**Figure 9:**
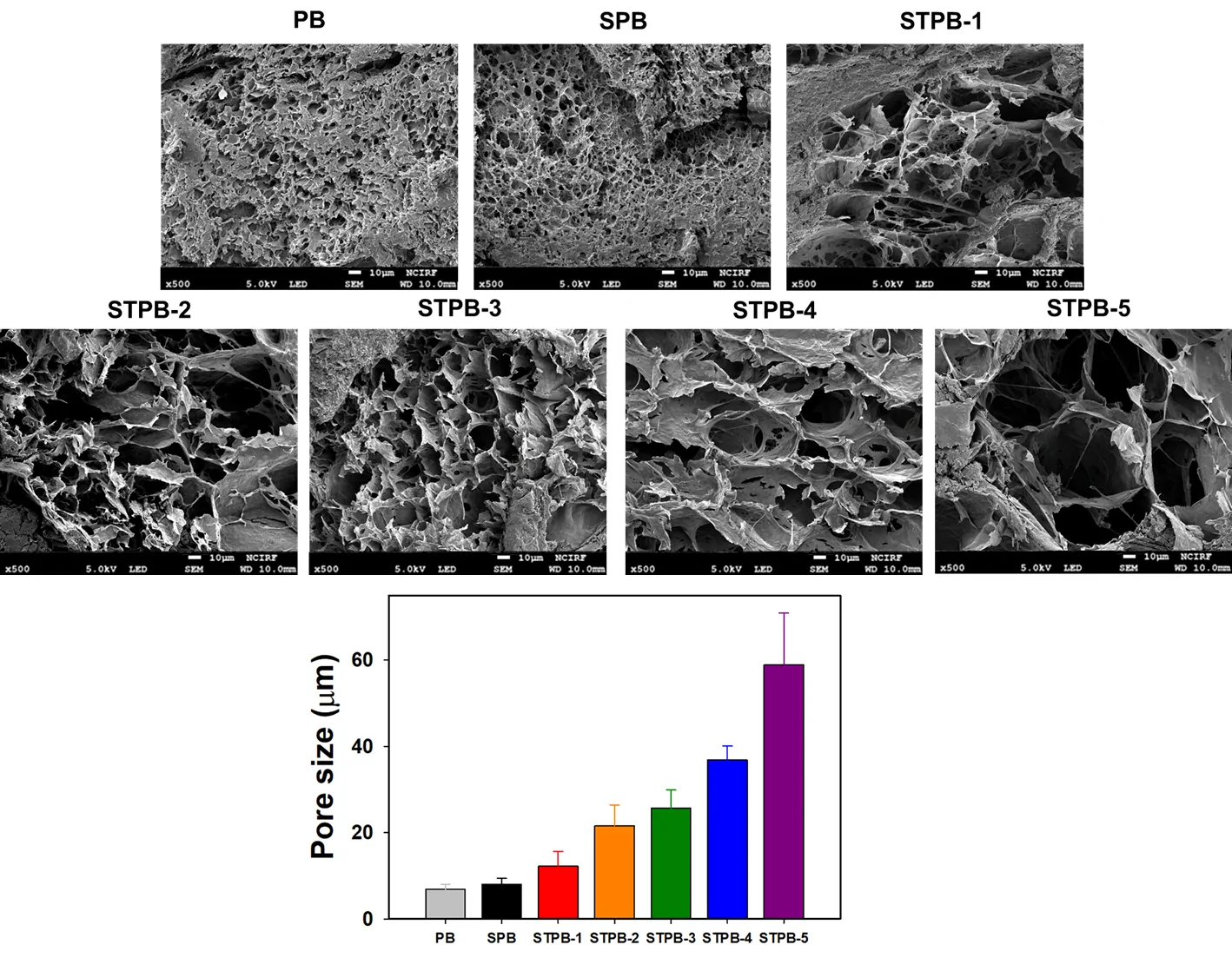
SEM images and apparent pore sizes of freeze-dried PB, SPB, and STPB hydrogels. Pore sizes were determined from 10 randomly selected pores per sample and are presented as mean ± SD. Scale bars are shown in each SEM image.

In Figure 9, the freeze-dried morphology of PB, SPB, and STPB hydrogels was examined by SEM. Because freezing and lyophilization can alter the apparent pore morphology of hydrogels, these SEM images are interpreted primarily as comparative morphologies of samples prepared under identical conditions rather than as direct representations of the hydrated network [69,70]. The PB hydrogel exhibited a relatively dense and compact structure with small pores. In contrast, SPB showed a more porous morphology, indicating the influence of SG incorporation. Quantitative image analysis showed mean apparent pore sizes of 6.82 ± 1.18, 7.95 ± 1.42, 12.23 ± 3.43, 21.60 ± 4.84, 25.69 ± 4.17, 36.83 ± 3.34, and 58.95 ± 11.98 μm for PB, SPB, and STPB-1–STPB-5, respectively. These composition-dependent morphological differences are consistent with previous reports showing that incorporation of polysaccharide- and polyphenol-containing components can alter the freeze-dried microstructure of dynamically crosslinked hydrogels [65,68,71]. Interestingly, the increase in apparent SEM pore size did not parallel the swelling behavior, which generally decreased with increasing TA-equivalent phenolic content of the incorporated SG-TA derivatives across the STPB series. Together with the rheological results, these observations are consistent with composition-dependent changes in matrix morphology, hydration behavior, and dynamic intermolecular interactions following SG-TA incorporation.

### 2.9. Deformability and Self-Healing Behavior of STPB Hydrogels

**Figure 10:**
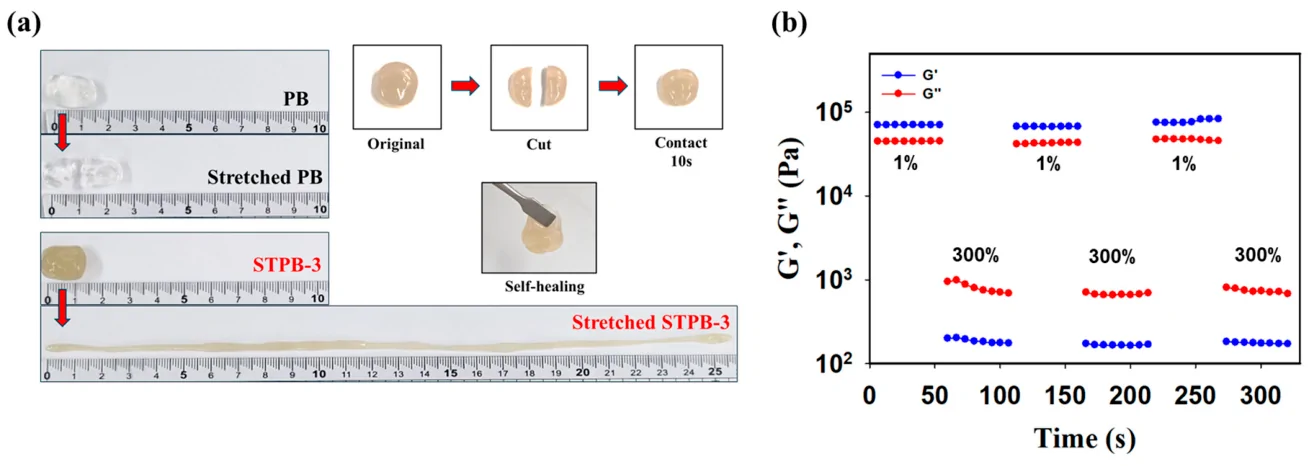
Macroscopic deformability and dynamic recovery behavior of STPB-3. (**a**) Stretchability comparison between PB and STPB-3 and macroscopic cut-and-rejoin sequence of STPB-3, showing rejoining within 10 s after direct contact of the cut surfaces. (**b**) Step-strain rheological recovery of STPB-3 under alternating strains of 1% and 300%.

The mechanical deformability and self-healing behavior of PB and STPB-3 hydrogels were evaluated [72]. The STPB-3 hydrogel exhibited marked stretchability, maintaining structural integrity under large deformation, whereas the PB hydrogel fractured easily under similar conditions (Figure 10a). In addition, after the freshly cut surfaces of STPB-3 were brought into direct contact, macroscopic rejoining occurred within 10 s without external stimulation. These results indicate that the incorporation of SG-TA is associated with enhanced deformability and rapid macroscopic rejoining of the dynamic hydrogel network [65,66]. The rheological recovery behavior was further evaluated by step-strain rheological measurements (Figure 10b). Under high strain, the storage modulus (G′) decreased below the loss modulus (G″), indicating disruption of the network structure. Upon returning to low strain, G′ recovered to 95.5% of its initial value during the first recovery interval. At the first measurable point after returning to 1% strain (approximately 10 s), G′ had already recovered to 95.8% of its initial value. Following the second high-strain cycle, the recovered G′ reached 110.6% of the initial value, indicating continued rearrangement of the reversible network during repeated deformation. These rapid and repeatable changes are consistent with the reconstruction of a dynamic network supported by reversible borate–diol interactions and secondary intermolecular interactions involving SG-TA [73].

### 2.10. Stimuli-Responsive 5-FU Release Behavior of STPB Hydrogel

5-fluorouracil (5-FU) was used as a model small-molecule drug to evaluate the responsiveness of the dynamic hydrogel network [74]. Prior to the release study, the actual drug-loading content and encapsulation efficiency of PB, SPB, and STPB-3 were determined (Table 2). Drug-loading contents were 8.89 ± 0.11%, 8.84 ± 0.24%, and 8.29 ± 0.04% for PB, SPB, and STPB-3, respectively, while the corresponding encapsulation efficiencies were 99.60 ± 1.21%, 97.49 ± 2.67%, and 99.56 ± 0.53%. These results showed high retention of 5-FU during hydrogel preparation. These experimentally determined drug contents were subsequently used to calculate cumulative 5-FU release.

**Figure 11:**
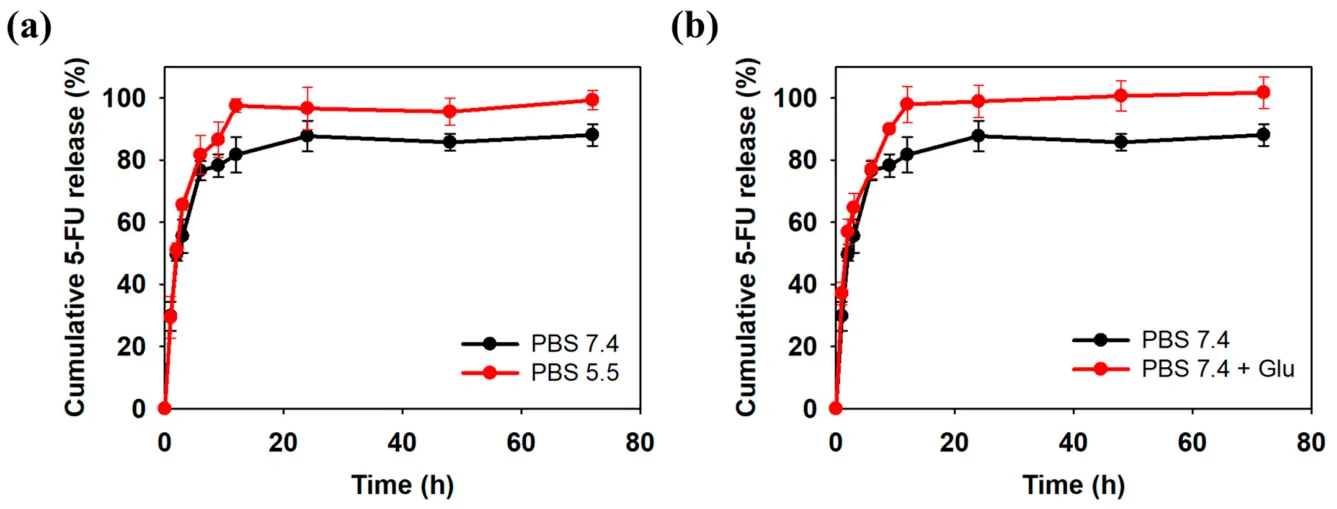
Stimuli-responsive 5-FU release behavior of STPB-3 hydrogel under (**a**) PBS at pH 7.4 and pH 5.5 and (**b**) PBS at pH 7.4 in the absence or presence of glucose (4 mg/mL). Data are presented as mean ± SD (*n* = 3).

In Figure 11, the release profiles of 5-FU from the STPB-3 hydrogel were evaluated to examine the stimuli-responsive drug release capability of the dynamic borate–diol network. At pH 7.4, the hydrogel showed a relatively moderate release profile. The cumulative release increased during the initial stage and reached approximately 78.2% at 9 h and 81.7% at 12 h, followed by a gradual increase to approximately 88.1% at 72 h. This profile indicated relatively gradual release under the pH 7.4 reference condition. Under acidic conditions (pH 5.5), the release was markedly accelerated. Approximately 51.0% of the drug was released within 2 h, increasing to 97.5% at 12 h and approximately 99.2% at 72 h. Compared with pH 7.4, the release was noticeably faster. This accelerated release is consistent with reduced stability of reversible borate–diol interactions under acidic conditions, which can promote network relaxation and facilitate 5-FU diffusion [75,76]. PBS at pH 5.5 was used as a representative acidic challenge condition for evaluating pH responsiveness.

In the presence of glucose, the release was also higher than that observed at pH 7.4. Approximately 97.9% of the loaded 5-FU was released within 12 h, and the cumulative release remained near complete thereafter. The enhanced release in glucose-containing medium is consistent with perturbation of the reversible borate–diol interaction environment by glucose through its cis-diol groups [77,78]. The glucose concentration of 4 mg/mL (approximately 22.2 mM) was used as a high-glucose challenge condition, comparable to concentrations employed as hyperglycemic stimuli in recent glucose-responsive hydrogel studies [79].

To further evaluate the influence of hydrogel composition on the release behavior, free 5-FU and 5-FU-loaded PB and SPB were examined under the same three conditions, and the corresponding profiles are presented in Figure S10. Free 5-FU rapidly dispersed into the release medium under all conditions. PB also showed relatively rapid release, reaching approximately 93.1%, 95.9%, and 93.2% at 12 h in PBS at pH 7.4, PBS at pH 5.5, and glucose-containing PBS, respectively, indicating only modest stimulus-dependent differences. In contrast, SPB showed a clearer pH-dependent response, with cumulative release at 12 h increasing from 79.6% at pH 7.4 to 91.9% at pH 5.5. In glucose-containing PBS, SPB reached 89.1% release at 12 h, showing a less pronounced glucose-dependent response than STPB-3. These composition-dependent differences indicate that incorporation of SG and SG-TA altered the stimulus-responsive release behavior of the PVA/borax-based network.

Post-release total drug recovery was 97.70 ± 1.32%, 99.82 ± 0.58%, and 97.69 ± 1.86% for PB, SPB, and STPB-3, respectively (Table 2), supporting satisfactory mass balance throughout the release experiments. The theoretical maximum 5-FU concentration in the 10 mL release medium was approximately 1 mg/mL upon complete release, substantially below its reported aqueous solubility of approximately 13 mg/mL [80]. In addition, replacement of each withdrawn aliquot with an equal volume of fresh medium further supported maintenance of sink conditions during the release study.

Kinetic fitting further showed that the preferred release model varied with formulation and release condition (Tables S1–S3). At pH 7.4, the Korsmeyer–Peppas model provided the best fit for all three hydrogel formulations, whereas first-order kinetics predominated under acidic conditions. Under glucose-containing conditions, PB and STPB-3 were best described by the first-order model, while SPB showed the highest fit to the Korsmeyer–Peppas model. Notably, SPB showed a less pronounced glucose-responsive release behavior than STPB-3. These results indicate that 5-FU release was governed by both hydrogel composition and environmental conditions.

Overall, the STPB hydrogel exhibited clear pH- and glucose-responsive drug release behavior. While release was relatively gradual under the pH 7.4 reference condition, it became accelerated under acidic and high-glucose conditions. These results suggest that the STPB hydrogel is a promising stimuli-responsive platform for model small-molecule delivery under different environmental conditions.

### 2.11. Antioxidant Activities of STPB Hydrogels

**Figure 12:**
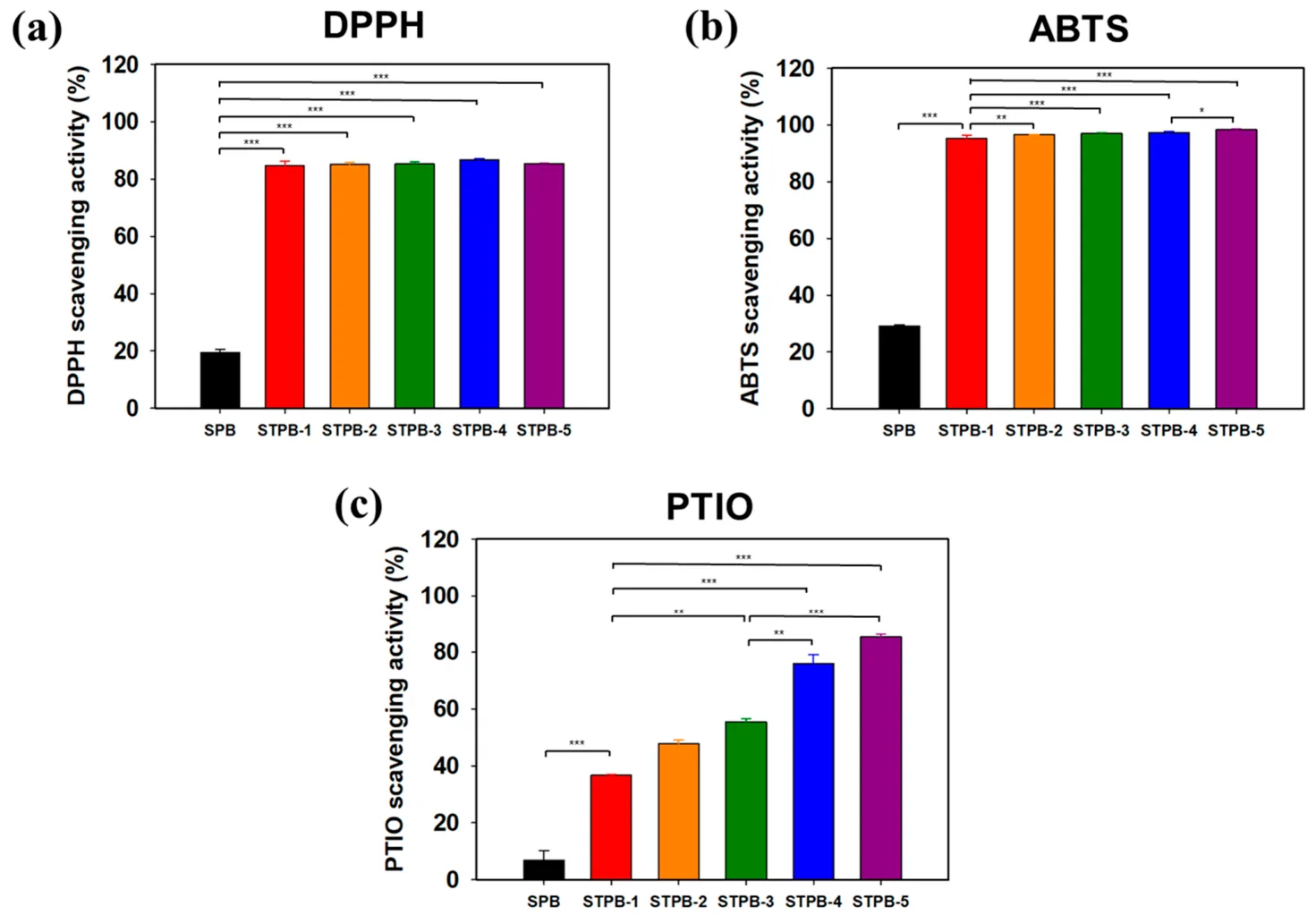
Antioxidant activities of PBS extracts from SPB and STPB hydrogels evaluated using (**a**) DPPH, (**b**) ABTS, and (**c**) PTIO radical-scavenging assays. Data are presented as mean ± SD (*n* = 3). Statistical significance was evaluated by one-way ANOVA followed by Tukey’s multiple-comparison test. * *p* < 0.05, ** *p* < 0.01, and *** *p* < 0.001.

The antioxidant activities of PBS extracts from SPB and STPB hydrogels were evaluated using DPPH, ABTS, and PTIO assays. All STPB samples exhibited markedly enhanced radical scavenging activity compared with SPB (Figure 12). In the DPPH and ABTS assays, STPB hydrogels showed consistently high scavenging activities of 84.7–86.5% and 95.2–98.4%, respectively (Figure 12a,b). Specifically, DPPH scavenging increased from 19.41 ± 1.16% for SPB to 84.67 ± 1.42% for STPB-1 and remained high across the STPB series, reaching 86.55 ± 0.53% for STPB-4. Similarly, ABTS scavenging increased from 29.09 ± 0.31% for SPB to 95.25 ± 0.86% for STPB-1 and reached 98.42 ± 0.09% for STPB-5. In the PTIO assay, STPB hydrogels also exhibited substantially higher scavenging activity than SPB, with values increasing from 36.74 ± 0.13% for STPB-1 to 85.31 ± 0.88% for STPB-5, compared with 6.88 ± 3.25% for SPB (Figure 12c). These results indicate that the antioxidant functionality of SG-TA was effectively retained after incorporation into the hydrogel network.

The high DPPH and ABTS scavenging activities demonstrate the substantial antioxidant capacity retained in the STPB system, while the PTIO assay reveals a more composition-dependent antioxidant response. The near-plateau DPPH and ABTS responses across STPB-1–STPB-5 suggest that the antioxidant capacity detectable by these assays was already high in the lower-TA formulations, whereas PTIO more clearly distinguished the effect of increasing TA-equivalent phenolic content across the STPB series. This behavior is consistent with contributions from SG-TA-derived phenolic functionality present in the hydrogel matrix and/or released into the PBS extract [81,82]. Overall, the STPB hydrogels exhibited robust antioxidant performance, demonstrating that SG-TA can serve as an effective bioactive component for hydrogels with antioxidant functionality originating from the SG-TA network-forming component.

### 2.12. Antibacterial Activity of STPB Hydrogels

**Figure 13:**
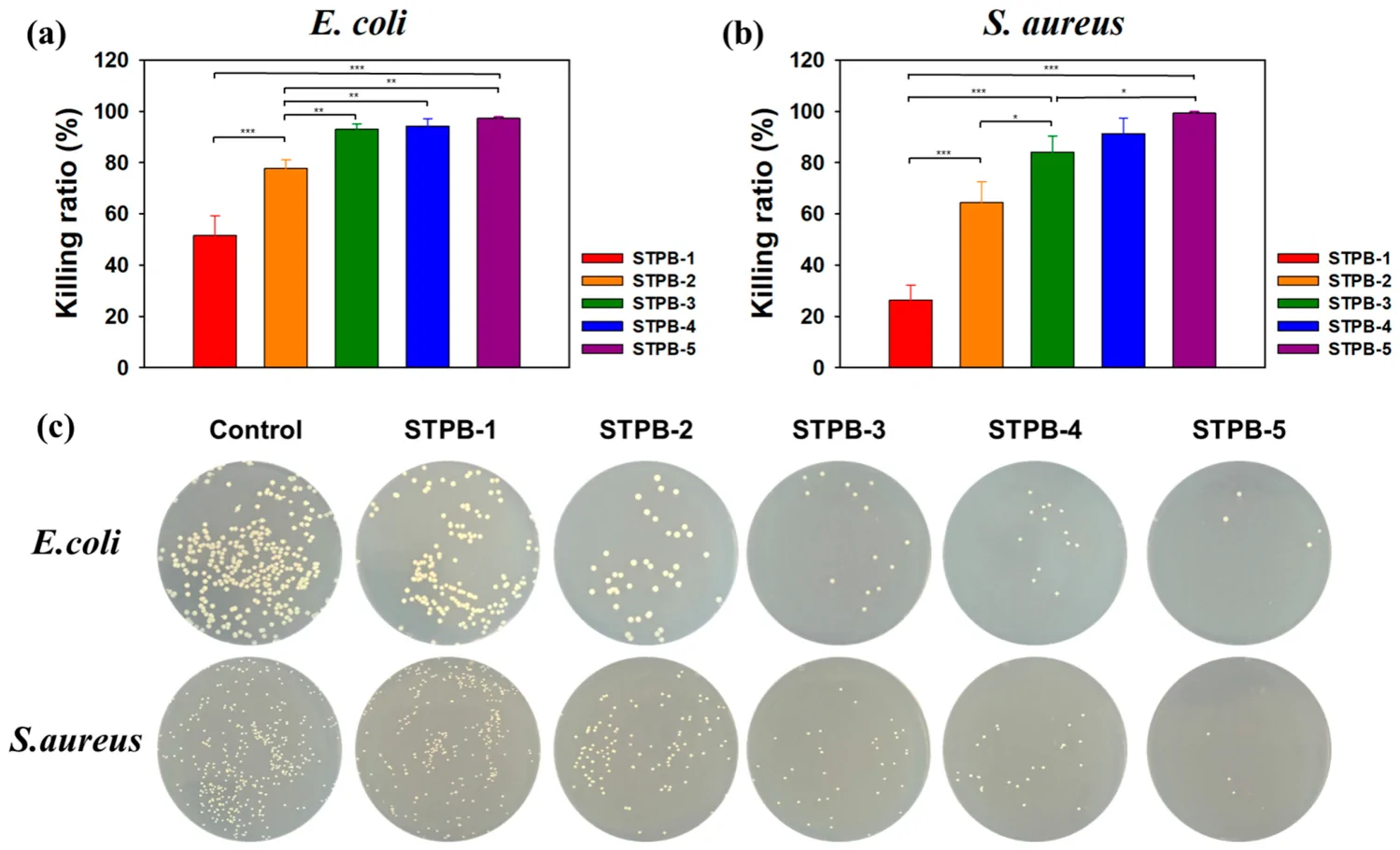
Antibacterial activities of PBS extracts from STPB hydrogels against (**a**) *E. coli* and (**b**) *S. aureus*, with (**c**) representative CFU images. Data are presented as mean ± SD (*n* = 3). Statistical significance was evaluated by one-way ANOVA followed by Tukey’s multiple-comparison test. * *p* < 0.05, ** *p* < 0.01, and *** *p* < 0.001.

In Figure 13, the antibacterial activity of STPB hydrogels was evaluated against *E. coli* and *S. aureus* using PBS extracts. As observed for SG-TA derivatives, the antibacterial activity of STPB hydrogels increased with increasing TA-equivalent content of the incorporated SG-TA derivatives. For *E. coli*, the killing ratio increased from 51.63 ± 7.52% for STPB-1 to 97.21 ± 0.62% for STPB-5, while for *S. aureus*, it increased from 26.23 ± 5.98% to 99.25 ± 0.57%. Notably, STPB-3 already exhibited killing ratios of 93.02 ± 2.11% against *E. coli* and 84.08 ± 6.34% against *S. aureus*, while STPB-4 and STPB-5 showed more than 90% killing against both bacterial strains. The progressive increase in antibacterial activity across the STPB series was consistent with the increasing TA-equivalent phenolic content of the incorporated SG-TA derivatives. Because PBS extracts were used for the assay, the observed antibacterial activity is likely associated with SG-TA-derived phenolic species present in the hydrogel extracts. These composition-dependent results suggest that the antibacterial performance of STPB hydrogels is closely associated with the incorporated SG-TA component. The SG-TA-derived phenolic species may contribute to antibacterial action through membrane perturbation, protein interactions, metal ion chelation, and related pathways reported for tannic acid-containing systems [76,83]. Overall, these results indicate that incorporation of SG-TA effectively transferred substantial antibacterial functionality to the hydrogel system. Considering the rheological and bioactivity results together, STPB-3 was selected as the representative formulation for subsequent self-healing and drug-release evaluation. While STPB-4 and STPB-5 exhibited the highest antibacterial activities, the antioxidant responses were already high across the STPB series, and the higher-TA formulations showed progressively lower viscoelastic moduli. STPB-3 therefore provided an intermediate composition that retained substantial antioxidant and antibacterial activity while maintaining higher viscoelastic moduli than STPB-4 and STPB-5.

### 2.13. Time-Dependent TA-Equivalent Phenolic Release from STPB Hydrogels

**Figure 14:**
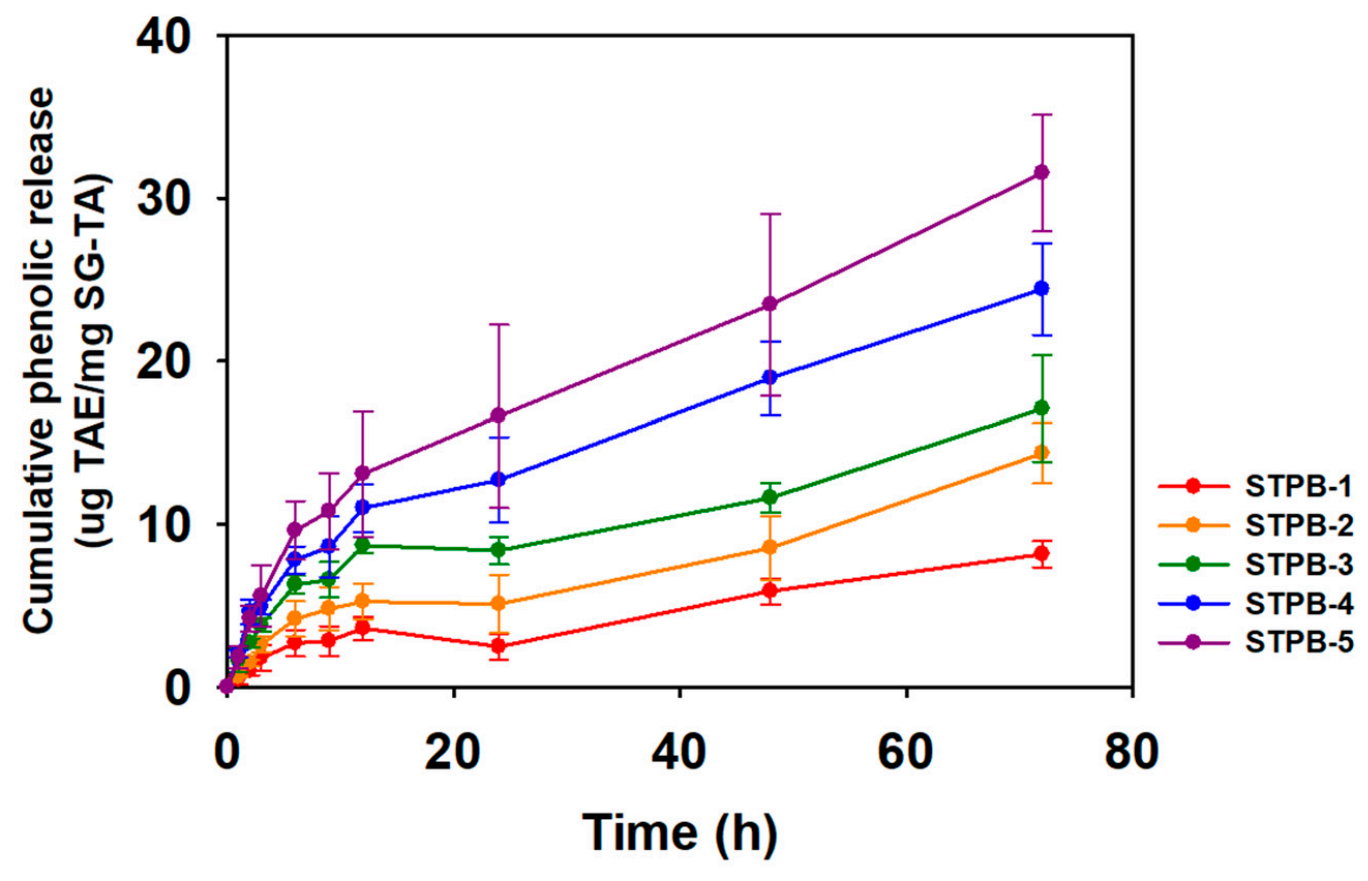
Time-dependent cumulative phenolic release from STPB hydrogels in PBS (pH 7.4), expressed as tannic acid equivalents (TAE) per initial SG-TA mass. Data are presented as mean ± SD (*n* = 3).

To further examine the contribution of releasable phenolic components to the bioactivity of the STPB hydrogels, the time-dependent cumulative phenolic release was evaluated over 72 h in PBS (pH 7.4) (Figure 14). Released phenolic content was expressed as tannic acid equivalents (TAE) and normalized to the initial mass of SG-TA incorporated into each hydrogel [84]. All STPB formulations exhibited measurable phenolic release, with the cumulative amount generally increasing with both incubation time and TA-equivalent content of the incorporated SG-TA derivatives. At 72 h, the cumulative phenolic release reached approximately 8.2, 14.4, 17.2, 24.5, and 31.5 µg TAE/mg SG-TA for STPB-1, STPB-2, STPB-3, STPB-4, and STPB-5, respectively. The progressively higher cumulative release from STPB-1 to STPB-5 was consistent with the increasing TA-equivalent phenolic content of the corresponding SG-TA derivatives [85]. The observed release demonstrates that a releasable fraction of SG-TA-derived phenolic components is present within the dynamic STPB network. Because SG-TA itself constitutes a polymeric component of the PVA/borax network rather than a separately loaded free tannic acid additive, the detected phenolic release may reflect gradual dissociation and diffusion of SG-TA-derived components from the hydrated dynamic network. Importantly, the composition-dependent phenolic release is consistent with the antioxidant and antibacterial activities observed for the corresponding hydrogel extracts, suggesting that released SG-TA-derived phenolic species contribute, at least in part, to the measured bioactivities. Thus, the bioactivity of the STPB system originates from the SG-TA network-forming component and can be expressed through both matrix-associated and releasable phenolic functionality [86].

### 2.14. Cytocompatibility of STPB Hydrogels

**Figure 15:**
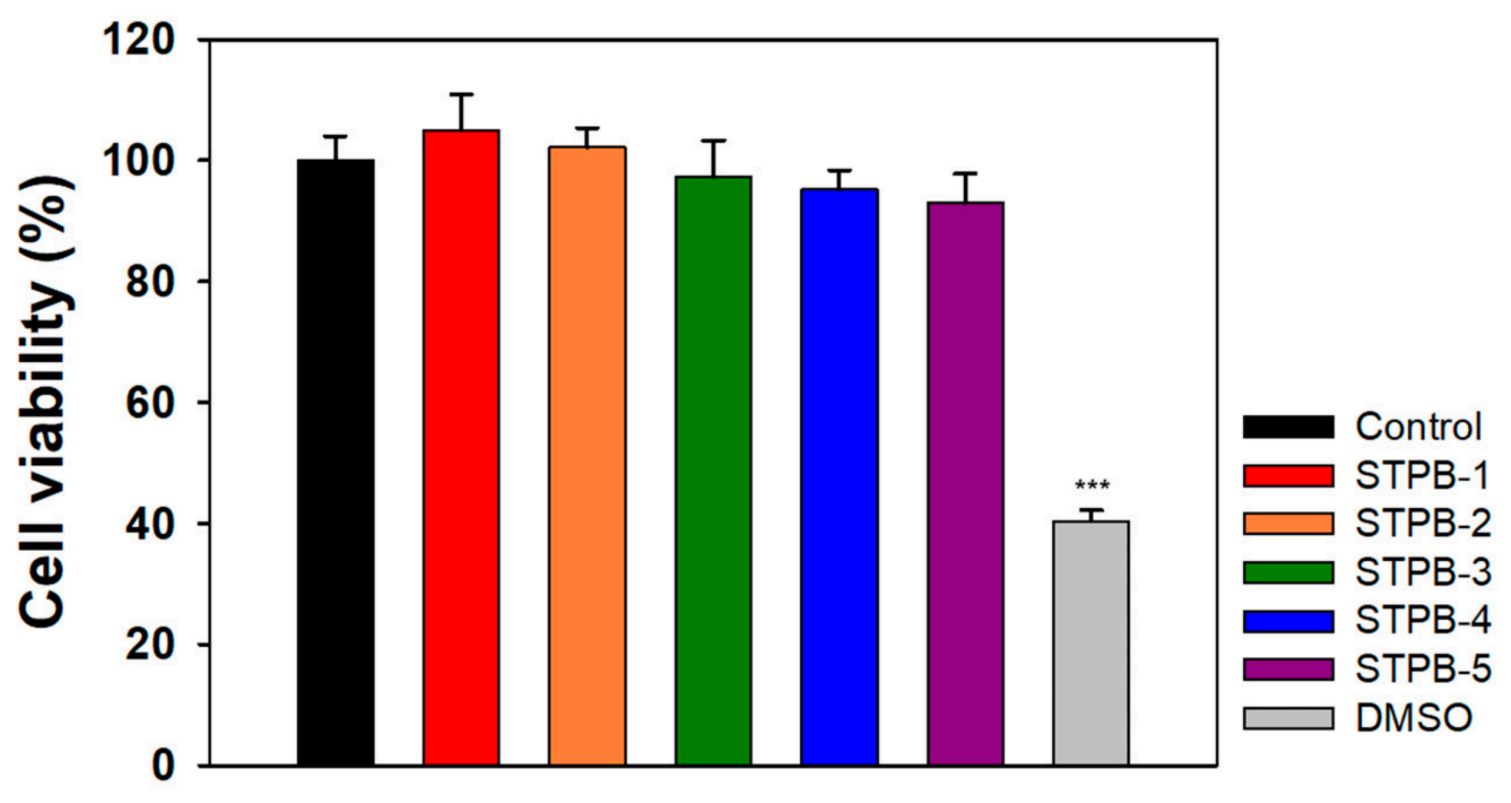
Cell viability of HEK-293 cells after 24 h treatment with STPB hydrogel extracts. DMSO was used as a positive cytotoxicity control. Data are presented as mean ± SD (*n* = 3). Statistical significance was evaluated by one-way ANOVA followed by Dunnett’s multiple-comparison test against the untreated control. *** *p* < 0.001.

The cytocompatibility of STPB hydrogels was evaluated using HEK-293 cells as a general mammalian cell model for preliminary in vitro cytocompatibility assessment (Figure 15) [87]. STPB-1–STPB-5 maintained high cell viabilities of 104.92 ± 6.00%, 102.15 ± 3.13%, 97.19 ± 6.12%, 95.14 ± 3.20%, and 92.93 ± 4.80%, respectively, after 24 h of treatment. None of the STPB-treated groups showed a significant decrease in cell viability relative to the untreated control. In contrast, the DMSO-treated positive control showed a markedly reduced cell viability of 40.40 ± 1.75% (*p* < 0.001). These results indicate favorable preliminary in vitro cytocompatibility of the STPB hydrogels toward HEK-293 cells under the tested conditions [10,88]. Together with their antioxidant, antibacterial, self-healing, and stimuli-responsive release properties, these findings support further evaluation of STPB hydrogels as multifunctional biomaterial platforms.

## 3. Conclusions

In this study, tannic acid-modified succinoglycan (SG-TA) was prepared through free-radical-mediated modification as an intrinsically bioactive microbial polysaccharide. Structural and physicochemical analyses supported the incorporation of TA-derived phenolic functionality into SG, resulting in markedly enhanced antioxidant and antibacterial activities. SG-TA was subsequently incorporated into a PVA/borax network to form dynamic STPB hydrogels exhibiting composition-dependent viscoelasticity, improved deformability, rapid rheological recovery, macroscopic self-rejoining, and preliminary cytocompatibility. The reversible network also enabled pH- and glucose-responsive release of 5-fluorouracil, with accelerated release under acidic and high-glucose conditions. Time-dependent phenolic-release analysis further demonstrated that SG-TA-derived phenolic functionality was expressed through both matrix-associated and releasable components, which likely contributed to the observed antioxidant and antibacterial activities. Overall, these findings demonstrate the potential of SG-TA as a bioactive network-forming component for multifunctional, self-healing, and stimuli-responsive hydrogel systems.

## 4. Materials and Methods

### 4.1. Materials and Reagents

For succinoglycan production, the *Sinorhizobium meliloti* Rm1021 strain was obtained from the Microbial Carbohydrate Resource Bank (MCRB), Konkuk University, Seoul, Republic of Korea. Ascorbic acid (99.7%), tannic acid, poly(vinyl alcohol) (PVA, M_w_ 89,000–98,000, 99% hydrolyzed), sodium tetraborate (borax), sodium carbonate, ABTS (≥99.0%), and potassium persulfate (≥99.0%) were purchased from Sigma-Aldrich (St. Louis, MO, USA). Hydrogen peroxide (30%) was purchased from Daejung Chemicals (Siheung, Republic of Korea). DPPH (95%) was purchased from Thermo Scientific (Waltham, MA, USA). PTIO (≥98.0%) was purchased from Tokyo Chemical Industry Co., Ltd. (Tokyo, Japan). Ethanol (99.9%) was purchased from Samchun Pure Chemicals (Pyeongtaek, Republic of Korea). DMEM (Fresh Media, high glucose) and FBS (Gold Serum) were purchased from Welgene (Gyeongsan, Republic of Korea).

### 4.2. Production and Purification of Succinoglycan (SG)

*Sinorhizobium meliloti* Rm1021 was cultured in Glutamate–Mannitol–Salts (GMS) medium containing D-mannitol (10 g/L), K_2_HPO_4_ (1.0 g/L), L-glutamic acid (1.0 g/L), MgSO_4_·7H_2_O (0.2 g/L), and CaCl_2_·2H_2_O (0.04 g/L). The pH of the medium was adjusted to 7.0 prior to sterilization. After autoclaving, a trace element solution was aseptically added. The culture was incubated at 30 °C for 7 days with shaking at 150 rpm. Following cultivation, bacterial biomass was separated by centrifugation at 8000× *g* for 15 min. The supernatant was concentrated under reduced pressure and mixed with cold ethanol at a ratio of 1:3 (*v*/*v*) to induce polysaccharide precipitation. The precipitate was collected and dialyzed against distilled water using a 12–14 kDa MWCO membrane for 3 days, with periodic replacement of the dialysis water. The purified SG solution was frozen and lyophilized to obtain SG powder. The purified SG was subsequently characterized by FTIR, ^1^H NMR, and UV–Vis spectroscopy, with UV–Vis analysis used for qualitative assessment of nucleic acid- and protein-related impurities.

### 4.3. Synthesis of Tannic Acid-Grafted Succinoglycan (SG-TA)

Tannic acid-grafted succinoglycan (SG-TA) was synthesized via a free radical grafting method using an H_2_O_2_/ascorbic acid redox system [89]. Succinoglycan powder (1 g) was dispersed in distilled water (100 mL) and stirred until completely dissolved. Ascorbic acid (0.108 g) and hydrogen peroxide solution (1 M, 2 mL) were added to the SG solution to initiate radical formation. The mixture was purged with nitrogen for 30 min. Tannic acid was then added at different feed ratios relative to SG (TA:SG = 0.2:1, 0.4:1, 0.6:1, 0.8:1, and 1:1, *w*/*w*, corresponding to 0.2, 0.4, 0.6, 0.8, and 1.0 g of TA, respectively). The grafting reaction was carried out at room temperature for 24 h under continuous stirring in a nitrogen atmosphere. The obtained reaction solution was purified by dialysis using a membrane with a molecular weight cut-off of 12–14 kDa. Dialysis was conducted against distilled water for 72 h, with periodic replacement of the external water to remove unreacted tannic acid and residual low-molecular-weight reagents. The dialyzed solution was frozen at −70 °C and lyophilized to obtain SG-TA powders. The resulting products were named SG-TA1 to SG-TA5 according to the initial tannic acid feed ratio. For comparison, an SG/TA physical-mixture control corresponding to the SG-TA3 composition was prepared under the same conditions but without addition of the H_2_O_2_/ascorbic acid redox initiator. The control mixture was subjected to the same dialysis and lyophilization procedures as the SG-TA samples. A redox-treated SG control (SG-redox) was prepared by subjecting SG to the same H_2_O_2_/ascorbic acid treatment and purification procedure used for SG-TA synthesis, but without tannic acid.

### 4.4. Preparation of SG-TA/PVA/Borax Hydrogels

Poly(vinyl alcohol) (PVA)-based hydrogels containing SG or SG-TA were prepared using borax as a crosslinker. To prepare the PVA stock solution, PVA was added to distilled water and stirred at 90 °C until a homogeneous 10% (*w*/*v*) solution was obtained. SG or SG-TA1–5 was then introduced into the PVA solution at 1% (*w*/*v*), and the mixture was heated again at 90 °C to obtain a uniform polymer solution. Hydrogel formation was induced by adding 0.30 mL of 2% (*w*/*v*) aqueous sodium tetraborate solution to 1.0 mL of the polymer mixture, followed by gentle mixing. The resulting formulation had a final volume of approximately 1.30 mL. For SPB and STPB formulations, each hydrogel contained 100 mg PVA, 10 mg SG or SG-TA, and 6 mg borax, corresponding to a PVA:polysaccharide:borax mass ratio of 100:10:6. PB was prepared without SG or SG-TA and contained 100 mg PVA and 6 mg borax. Gelation was achieved within several seconds at room temperature. Unless otherwise specified, the hydrogels were allowed to equilibrate at room temperature for 30 min before characterization. PB, SPB, and STPB denote PVA/borax, SG/PVA/borax, and SG-TA/PVA/borax hydrogels, respectively, and STPB-1 to STPB-5 correspond to hydrogels prepared with SG-TA1 to SG-TA5. The detailed compositions and final concentrations of the hydrogel formulations are summarized in Table 3.

**Table 3:** Composition, final concentrations, and formulation ratios of PB, SPB, and STPB hydrogels.

	Polysaccharide Component	PVA (% *w*/*v*)	Polysaccharide (% *w*/*v*)	Borax (% *w*/*v*)	Mass Ratio ^a^	Final Volume (mL)
PB	–	7.69	–	0.46	100:0:6	1.3
SPB	SG	7.69	0.77	0.46	100:10:6	1.3
STPB-1	SG-TA1	7.69	0.77	0.46	100:10:6	1.3
STPB-2	SG-TA2	7.69	0.77	0.46	100:10:6	1.3
STPB-3	SG-TA3	7.69	0.77	0.46	100:10:6	1.3
STPB-4	SG-TA4	7.69	0.77	0.46	100:10:6	1.3
STPB-5	SG-TA5	7.69	0.77	0.46	100:10:6	1.3

^a^ Mass ratio represents PVA:SG(SG-TA):borax.

### 4.5. Determination of Tannic Acid-Equivalent Phenolic Content

The tannic acid-equivalent (TAE) phenolic content in SG-TA samples was determined using the Folin–Ciocalteu colorimetric assay [38]. In each assay, 1 mL of SG-TA solution at 0.3 mg/mL was treated with 2.5 mL of 10% Folin–Ciocalteu reagent for 3 min. Subsequently, 7.5% (*w*/*v*) sodium carbonate solution (2 mL) was introduced to develop the color reaction. Color development was carried out for 1 h at room temperature under dark conditions, followed by absorbance measurement at 765 nm using a UV-2450 spectrophotometer (Shimadzu Corporation, Kyoto, Japan). The phenolic content was quantified using a calibration curve prepared with tannic acid standards and expressed as mg tannic acid equivalents (TAE) per g of recovered product. The recovered product yield was calculated from the mass of the lyophilized purified product relative to the combined initial masses of SG and TA. The amount of TA equivalents retained in the recovered product was estimated from the measured TAE content and the recovered product mass. Apparent grafting efficiency was then calculated as the ratio of the estimated retained TA equivalents to the initial TA feed, and the apparent unretained TA fraction was estimated by mass balance as 100% minus the apparent grafting efficiency.

(1) $$Recoverd\;product\;yield\left(\%\right)=\frac{m_{product}}{m_{SG}+m_{TA}}\times 100$$

(2) $$Apparent\;grafting\;efficiency\left(\%\right)=\frac{C_{TAE}\times m_{product}}{m_{TA}}\times 100$$

(3) $$Apparent\;unretained\;TA\left(\%\right)=100-Apparent\;grafting\;efficiency\;\left(\%\right)$$

where m*_product_* is the mass of the recovered lyophilized SG-TA product, m*_SG_* is the initial SG mass, m*_TA_* is the initial TA feed mass, and C*_TAE_* is the TA-equivalent phenolic content of the recovered product.

### 4.6. Molecular Weight Analysis

The apparent molecular weights of SG and SG-TA samples were estimated by gel permeation chromatography (GPC). SG and SG-TA samples were dissolved in distilled water at 0.5% (*w*/*v*) before injection. GPC was conducted using a Waters Breeze system coupled with a Waters 1525 binary pump and a Waters 2414 refractive index detector (Waters Corporation, Milford, MA, USA). Chromatographic separation was performed using Waters Ultrahydrogel Linear, 500, 250, and 120 columns (Waters Corporation, Milford, MA, USA) connected in series. A 0.02 N sodium nitrate solution was used as the mobile phase and delivered at 0.8 mL min^−1^. The apparent molecular weights were estimated using a calibration curve generated from pullulan standards covering a molecular weight range of 2.2 × 10^3^ to 6.42 × 10^5^ Da. Because pullulan standards were used for calibration, the obtained molecular weights are reported as apparent molecular weights rather than absolute molecular weights. Molecular-weight distributions and dispersity values were obtained from the corresponding GPC chromatograms.

### 4.7. Nuclear Magnetic Resonance Spectroscopy (NMR) Analysis

The structural characteristics of SG, SG-TA derivatives, and tannic acid were examined by ^1^H and ^13^C NMR spectroscopy. ^1^H NMR spectra were obtained for SG, SG-TA1–SG-TA5, tannic acid, and the SG/tannic acid physical-mixture control before and after dialysis. ^13^C NMR spectra were obtained for SG, SG-TA3, and tannic acid. Samples were reconstituted in deuterated water (D_2_O, 99.96%) at concentrations of 10 mg/mL for ^1^H NMR and 15 mg/mL for ^13^C NMR. Each solution (1 mL) was transferred into a 5 mm NMR tube, and spectra were recorded at 25 °C using a Bruker Avance 600 MHz spectrometer (Bruker BioSpin GmbH & Co. KG, Ettlingen, Germany).

### 4.8. Fourier Transform Infrared Spectroscopy (FTIR) Analysis

Infrared spectra were collected to analyze functional-group characteristics of SG, SG-TA derivatives, and the corresponding hydrogel formulations. Dried SG and SG-TA samples, as well as lyophilized PB, SPB, and STPB hydrogels, were analyzed using a Spectrum Two FTIR spectrometer equipped with an attenuated total reflectance accessory (PerkinElmer, Waltham, MA, USA). For each sample, spectra were collected over the range of 4000–650 cm^−1^ at a resolution of 2 cm^−1^, and 16 scans were averaged.

### 4.9. Rheological Analysis

The rheological properties of SG, SG-TA derivatives, and hydrogels were characterized using a DHR-2 rheometer (TA Instruments, New Castle, DE, USA).

For solution-state rheological measurements, SG and SG-TA derivatives were prepared as 1% (*w*/*v*) aqueous solutions and measured at 25 °C with 40 mm parallel-plate geometry. Viscosity measurements were performed under a shear rate ramp of 0.1–1000 s^−1^. Amplitude-sweep measurements were conducted from 0.1 to 1000% strain at an angular frequency of 1.0 rad/s to investigate the strain-dependent rheological behavior of SG and SG-TA derivatives. The redox-treated SG control was evaluated under the same shear-viscosity measurement conditions for comparison.

For hydrogel rheological measurements, PB, SPB, and STPB hydrogels were examined at 25 °C using 20 mm parallel plates. Frequency-sweep tests were conducted at a fixed strain of 0.5% while varying the angular frequency from 0.01 to 100 rad/s. The loss tangent (tan δ) was calculated as G″/G′ from the corresponding frequency-sweep data.

Hydrogel amplitude-sweep measurements were conducted from 0.1 to 1000% strain at an angular frequency of 1.0 rad/s to assess the strain-dependent mechanical response and network stability. Time-sweep measurements were additionally performed for freshly prepared PB, SPB, and STPB-3 immediately after mixing to monitor the time-dependent evolution of G′ and G″ during network formation at a fixed strain of 1% and an angular frequency of 1.0 rad/s for 140 s.

Self-healing behavior was further evaluated by step-strain measurements under alternating strains of 1% and 300% at an angular frequency of 10 rad/s. Each strain level was maintained for 50 s, with a 6 s interval between successive strain steps. The G′ recovery ratio was calculated from the mean G′ value during the recovered low-strain interval relative to the mean G′ value during the initial low-strain interval.

### 4.10. X-Ray Diffraction (XRD) Measurements

The structural ordering of SG and SG-TA samples was examined by XRD. Diffraction profiles were obtained with a Rigaku SmartLab diffractometer (Rigaku Corporation, Akishima, Japan) using Cu Kα radiation under operating conditions of 30 kV and 20 mA. The scanning range was set from 5° to 80° in 2θ.

### 4.11. Thermogravimetric Analysis (TGA) Measurements

The thermal degradation behavior of SG and SG-TA samples was investigated using an STA 449 F3 Jupiter thermogravimetric analyzer (NETZSCH-Gerätebau GmbH, Selb, Germany). Approximately 5 mg of dried sample was used for each measurement. Measurements were carried out under nitrogen by increasing the temperature from 30 to 600 °C at 10 °C min^−1^, and the corresponding weight loss was recorded during heating. Derivative thermogravimetric (DTG) curves were obtained from the TGA data to determine the maximum degradation temperature (T_max_).

### 4.12. Macroscopic Gelation, Stability, and Swelling Measurements

The minimum gel-forming concentration was evaluated by the vial-inversion method using formulations prepared with PVA concentrations ranging from 1 to 10% (*w*/*v*) under otherwise identical formulation conditions. After preparation, the samples were allowed to equilibrate at room temperature and the vials were inverted to assess whether the formulations maintained a self-supporting state without observable flow.

For short-term macroscopic stability evaluation, freshly prepared PB, SPB, and STPB-3 hydrogels were maintained at room temperature and visually examined over 24 h. Representative photographs were recorded to compare changes in macroscopic form.

For swelling measurements, lyophilized PB, SPB, and STPB hydrogel samples were weighed to obtain their initial dry mass and immersed in 10 mL of PBS (pH 7.4) at room temperature. At predetermined time points over 72 h, the samples were removed from the medium, gently blotted with filter paper to remove excess surface liquid, and immediately weighed. The swelling ratio was calculated as follows:

(4) $$Swelling\;ratio\left(\%\right)=\frac{W_{t}-W_{d}}{W_{d}}\;\times 100$$

where *W_d_* is the initial dry mass of the lyophilized hydrogel and *W_t_* is the mass of the swollen hydrogel at time t. Measurements were performed using three independently prepared samples (*n* = 3).

### 4.13. Morphological Analysis of PB, SPB, and STPB Hydrogels

The internal pore morphology of PB, SPB, and STPB hydrogels was observed by field emission scanning electron microscopy (FE-SEM) using a JSM-7800F Prime microscope (JEOL Ltd., Akishima, Tokyo, Japan). The hydrogel samples were frozen, lyophilized, and sectioned to expose their cross sections. The samples were attached to double-sided carbon tape and coated with a thin conductive layer by sputtering. FE-SEM images were obtained at 5 kV with a magnification of 500×. Apparent pore sizes were quantified from the SEM images using ImageJ software (version 1.54g). Ten pores were randomly selected from each sample, and the measured apparent pore sizes were expressed as mean ± SD.

### 4.14. Macroscopic Stretchability and Self-Healing Test

The macroscopic stretchability and self-healing behavior of PB and STPB-3 hydrogels were examined by manual stretching and cutting–rejoining tests. For the stretchability test, PB and STPB-3 hydrogels were manually stretched, and their deformation behavior was photographed. For the self-healing test, STPB-3 was cut into two separate pieces using a blade, and the freshly exposed cut surfaces were brought into direct contact without any external stimulus. After 10 s of contact, the rejoined sample was photographed to evaluate macroscopic self-rejoining behavior.

### 4.15. In Vitro 5-FU Release Assay

The release profiles of 5-fluorouracil (5-FU) were monitored at 37 °C under different pH and glucose conditions. Drug-loaded PB, SPB, and STPB-3 hydrogels were prepared by incorporating 10 mg of 5-FU into 1.0 mL of the corresponding polymer mixture prior to addition of 0.30 mL of borax solution. PBS at pH 7.4 and pH 5.5 was used to evaluate pH-responsive release, while PBS at pH 7.4 containing glucose (4 mg/mL) was used to evaluate glucose-responsive release. For the free-drug control, 10 mg of 5-FU was directly added to 10 mL of the corresponding release medium and subjected to the same sampling and medium-replacement procedure.

Prior to release analysis, the drug-loading content and encapsulation efficiency of PB, SPB, and STPB-3 were determined using the experimentally measured amount of 5-FU retained in each formulation.

To determine the amount of 5-FU retained in each formulation, the drug-loaded hydrogel was lyophilized and dispersed in 10 mL of distilled water. The resulting suspension was sonicated for 30 min and then continuously stirred for 24 h to extract the retained 5-FU. The 5-FU concentration was quantified by UV–Vis spectroscopy at 265 nm using a UV-2450 spectrophotometer (Shimadzu Corporation, Kyoto, Japan) and a 5-FU calibration curve. Corresponding drug-free PB, SPB, and STPB-3 hydrogels were processed in parallel under the same extraction conditions, and their absorbance at 265 nm was subtracted from that of the corresponding drug-loaded samples to correct for background absorbance from the hydrogel components. The experimentally determined amount was used for calculation of drug-loading content and encapsulation efficiency. Drug-loading content and encapsulation efficiency were calculated as follows:

(5) $$Drug\;loading\left(\%\right)=\frac{A}{B}\;\times 100$$

(6) $$Encapsulation\;efficiency\left(\%\right)=\frac{A}{C}\times 100$$

where *A* is the experimentally determined amount of 5-FU retained in the hydrogel, *B* is the dry mass of the drug-loaded hydrogel, and *C* is the initial 5-FU feed.

Each drug-loaded hydrogel was immersed in 10 mL of release medium. At predetermined time points, 1 mL of the release medium was withdrawn and immediately replaced with an equal volume of fresh medium. The released 5-FU concentration was quantified by UV–Vis absorbance at 265 nm. Corresponding drug-free PB, SPB, and STPB-3 hydrogels were examined in parallel under each release condition, and the absorbance of the time-matched blank was subtracted before conversion of absorbance to 5-FU concentration.

(7) $$Cumulative\;5-FU\;release\left(\%\right)=\frac{V_{0}C_{n}+V_{s}\sum_{i=1}^{n-1}C_{i}}{M_{0}}\times 100$$

where *V*_0_ is the total volume of release medium, *V_s_* is the sampling volume, *C_n_* is the 5-FU concentration at each sampling time, *C_i_* is the 5-FU concentration in the previously collected samples, and *M_0_* is the experimentally determined amount of 5-FU loaded in each hydrogel formulation.

The release profiles were further analyzed using zero-order, first-order, Higuchi, and Korsmeyer–Peppas kinetic models. Model fitting was evaluated using the coefficient of determination (R^2^). For the Korsmeyer–Peppas model, only data within the initial release region (Mt/M∞ ≤ 0.6) were used for fitting.

After completion of the release experiment, residual 5-FU remaining in the hydrogel was quantified using the same extraction and UV–Vis quantification procedure described above for determination of the retained 5-FU amount. Post-release total drug recovery was calculated as follows:

(8) $$Total\;drug\;recovery\left(\%\right)=\frac{D+E}{A}\;\times 100$$

where A is the experimentally determined amount of 5-FU retained in the hydrogel, *D* is the cumulative amount of released 5-FU and *E* is the residual amount of 5-FU remaining in the hydrogel after the release experiment.

### 4.16. Antioxidant Activity Assays

The radical scavenging activities of SG, SG-TA, and PBS extracts from SPB and STPB hydrogels were examined using DPPH, ABTS, and PTIO assays [90,91]. For the concentration-dependent DPPH and ABTS assays, SG and SG-TA samples were prepared in distilled water at 20–100 μg/mL, whereas concentrations of 0.2–1.0 mg/mL were used for the PTIO assay. The SG-TA3 physical-mixture control and free tannic acid were evaluated under the same conditions as the SG-TA samples. For SPB and STPB hydrogels, PBS extracts were prepared by immersing each hydrogel sample in 5 mL of PBS for 15 h at room temperature.

For the DPPH assay, an ethanolic DPPH solution (0.2 mM) was used as the radical source. The sample and DPPH solutions were combined in equal volumes and incubated for 30 min at 37 °C without light exposure. The absorbance was then recorded at 517 nm using a UV-2450 spectrophotometer (Shimadzu Corporation, Kyoto, Japan), and the scavenging activity was determined relative to the untreated radical control.

In the ABTS assay, ABTS radicals were generated by reacting 7 mM ABTS solution with 2.45 mM potassium persulfate solution for 12–16 h in the dark. Before use, the ABTS radical solution was diluted with distilled water to an absorbance of approximately 0.7 at 734 nm. The diluted ABTS radical solution (800 μL) was mixed with 200 μL of sample solution, and the reaction was allowed to proceed for 6 min at room temperature under dark conditions. After incubation, the absorbance was measured at 734 nm using a UV–Vis spectrophotometer.

For the PTIO assay, a PTIO radical solution was prepared in distilled water at 0.2 mg/mL. The sample and PTIO solutions were mixed at the same volume ratio and incubated for 2 h at room temperature in the dark. Following incubation, the reaction mixture was analyzed by measuring its absorbance at 557 nm with a UV–Vis spectrophotometer. Radical scavenging activity was calculated according to the following equation:

(9) $$Radical\;scavenging\;activity\left(\%\right)=\left[1-\frac{A_{sample}-A_{blank}}{A_{control}}\right]\;\times 100$$

where *A_sample_* is the absorbance of the sample solution mixed with the radical solution, *A_blank_* is the absorbance of the sample solution without the radical reagent, and *A_control_* is the absorbance of the control solution containing the radical reagent without the sample. All measurements were performed in triplicate.

### 4.17. Antibacterial Activity Assay

A plate counting assay was used to determine the antibacterial activity of SG, SG-TA, and PBS extracts from STPB hydrogels against *Escherichia coli* (ATCC 25922) and *Staphylococcus aureus* (ATCC 25923) [92]. SG and SG-TA samples were prepared as aqueous solutions at 10 mg/mL. For STPB hydrogels, extracts were obtained by immersing each hydrogel in 5 mL of PBS (pH 7.4) and incubating for 15 h. The collected extracts were used for antibacterial testing. *E. coli* and *S. aureus* were grown in LB medium at 37 °C. The resulting bacterial cultures were diluted to approximately 1 × 10^6^ CFU/mL before use. Equal volumes of bacterial suspension and each test sample, including SG solution, SG-TA solution, and hydrogel extract, were combined and incubated at 37 °C for 4 h. After incubation, the mixtures were serially diluted to 10^−4^ with LB medium. The diluted suspensions were plated on agar and cultured for 15 h at 37 °C. After incubation, colony numbers were recorded, and antibacterial activity was expressed as the decrease in viable colonies relative to the untreated control. The killing ratio was calculated using the following equation:

(10) $$Killing\;ratio\left(\%\right)=\left(\frac{N_{control}-N_{sample}}{N_{control}}\right)\times 100$$

where *N_control_* is the number of viable colonies in the untreated control group and *N_sample_* is the number of viable colonies after treatment with the SG, SG-TA, or hydrogel extract sample. Each experiment was conducted in triplicate.

### 4.18. Time-Dependent Phenolic Release from STPB Hydrogels

The time-dependent release of phenolic components from STPB hydrogels was evaluated in PBS (pH 7.4) over 72 h at 37 °C. Each STPB hydrogel was immersed in 10 mL of PBS (pH 7.4), and 1 mL aliquots of the release medium were collected at predetermined time points and replaced with an equal volume (1 mL) of fresh PBS. The released phenolic content was quantified at 275 nm using a UV-2450 UV–Vis spectrophotometer (Shimadzu Corporation, Kyoto, Japan) with a tannic acid calibration curve and expressed as tannic acid equivalents (TAE). Corresponding SPB samples were analyzed in parallel as time-matched blanks, and their absorbance was subtracted before quantification. Cumulative phenolic release was calculated with correction for the amount removed during previous sampling according to:

(11) $$Q_{n}=V_{0}C_{n}+V_{s}\sum_{i=1}^{n-1}C_{i}$$

where *Q_n_* is the cumulative amount of released phenolic content at sampling time *n*, *V*_0_ is the initial release-medium volume, *V_s_* is the sampling volume, *C_n_* is the TAE concentration at sampling time *n*, and *C*_i_ represents the TAE concentrations in the previously collected samples. The cumulative phenolic release was normalized to the initial mass of SG-TA incorporated into each hydrogel and expressed as µg TAE/mg SG-TA. All measurements were performed in triplicate.

### 4.19. In Vitro Cell Viability Assay

An MTT assay was performed to examine the cytocompatibility of STPB hydrogels toward human embryonic kidney 293 (HEK-293) cells obtained from the Korean Cell Line Bank (KCLB; Seoul, Republic of Korea) [93]. Hydrogel-treated DMEM was prepared by incubating 1 g of STPB hydrogel in 5 mL of DMEM for 15 h prior to cell treatment. Cells were cultured in DMEM containing 10% fetal bovine serum and 1% penicillin–streptomycin in a humidified incubator at 37 °C with 5% CO_2_. For the cell viability assay, HEK-293 cells were cultured for 15 h in a 96-well plate to allow cell attachment. The culture medium was then replaced with hydrogel-treated DMEM. Fresh DMEM served as the untreated control, whereas DMSO-containing DMEM served as the positive cytotoxicity control. After 24 h of treatment, MTT reagent solution was applied to the cells, and the cells were left for 4 h to form formazan crystals. The MTT reagent was then removed, and solubilization buffer was added to dissolve the formazan precipitate for 15 h. The optical density of each well was recorded at 540 nm using a SpectraMax Microplate Reader (Molecular Devices, Sunnyvale, CA, USA), and relative cell viability was calculated as follows:

(12) $$Cell\;viability\;\left(\%\right)=\frac{A_{sample}}{A_{control}}\;\times 100$$

where *A_sample_* is the absorbance of cells treated with hydrogel-treated DMEM and *A_control_* is the absorbance of cells cultured in the untreated control medium. All experiments were performed in triplicate.

### 4.20. Statistical Analysis

Quantitative data are presented as mean ± standard deviation (SD). Unless otherwise specified, experiments were performed using three independently prepared samples (*n* = 3). Statistical comparisons among multiple groups were performed using one-way analysis of variance (ANOVA) followed by Tukey’s multiple-comparison test. For the cell viability assay, Dunnett’s multiple-comparison test was used to compare each treatment group with the untreated control. Statistical significance was defined as *p* < 0.05, with * *p* < 0.05, ** *p* < 0.01, and *** *p* < 0.001. For SEM pore-size analysis, ten randomly selected pores per sample were measured and the results are presented as mean ± SD; these measurements were not treated as independent hydrogel replicates.

## Figures and Tables

**Table 1:** Tannic acid feed, recovery, TA-equivalent phenolic content, apparent grafting efficiency, unretained TA and apparent molecular weight of SG and SG-TA derivatives.

	TA Feed (mg)	Recovered Product Yield (%) ^a^	TA-Equivalent Phenolic Content (mg TAE/g Product)	Apparent Grafting Efficiency (%) ^b^	Unretained TA (%) ^c^	Apparent M_w_ (Da)
SG	–	–	–	–	–	324,264
SG-TA1	200	68.21	96.50 ± 0.49	39.49 ± 0.20	60.51 ± 0.20	348,605
SG-TA2	400	70.12	134.64 ± 2.91	33.04 ± 0.71	66.96 ± 0.71	360,625
SG-TA3	600	56.75	230.03 ± 2.15	34.81 ± 0.33	65.19 ± 0.33	352,775
SG-TA4	800	52.42	262.12 ± 1.86	30.92 ± 0.22	69.08 ± 0.22	379,066
SG-TA5	1000	47.89	321.97 ± 3.40	30.84 ± 0.33	69.16 ± 0.33	336,283

^a^ Relative to the combined initial masses of SG and TA. ^b^ Calculated on a TAE basis relative to the initial TA feed. ^c^ Estimated on a TAE basis as the fraction of the initial TA feed not accounted for in the recovered product. TAE, tannic acid equivalent. Apparent average molecular weight (M_w_) was determined by GPC using pullulan standards.

**Table 2:** Drug-loading content, encapsulation efficiency, and post-release total drug recovery of 5-FU-loaded hydrogel formulations. Data are presented as mean ± SD (*n* = 3).

	Drug Loading (%)	Encapsulation Efficiency (%)	Total Drug Recovery (%)
PB	8.89 ± 0.11	99.60 ± 1.21	97.70 ± 1.32
SPB	8.84 ± 0.24	97.49 ± 2.67	99.82 ± 0.58
STPB-3	8.29 ± 0.04	99.56 ± 0.53	97.69 ± 1.86

## Data Availability

The original contributions presented in this study are included in the article. Further inquiries can be directed to the corresponding author.

## Supplementary Materials

The following supporting information can be downloaded at https://www.mdpi.com/article/10.3390/gels12090828/s1: Figure S1: Additional characterization of purified SG: (a) UV–Vis absorbance spectrum, (b) FTIR spectrum, and (c) ^1^H NMR spectrum; Figure S2: GPC chromatograms and molecular-weight distribution parameters of SG and SG-TA1–SG-TA5; Figure S3: 1H NMR spectra of SG and SG-TA derivatives prepared with different tannic acid feed ratios; Figure S4: 1H NMR spectra of the SG/tannic acid physical mixture (a) before and (b) after dialysis; Figure S5: Amplitude-sweep rheological profiles of SG and SG-TA1–SG-TA5 solutions; Figure S6: Effect of H_2_O_2_/ascorbic acid redox treatment on the shear viscosity of SG; Figure S7: TGA curves of SG and SG-TA1–SG-TA5; Figure S8: Frequency-dependent loss tangent (tan δ) of PB, SPB, and STPB hydrogels; Figure S9: Time-sweep rheological profiles of PB, SPB, and STPB-3 hydrogels; Figure S10: 5-FU release profiles of control formulations under different release conditions: 5-FU-loaded PB, 5-FU-loaded SPB, and free 5-FU; Table S1: Kinetic-model fitting parameters for 5-FU release from PB, SPB, and STPB-3 in PBS at pH 7.4; Table S2: Kinetic-model fitting parameters for 5-FU release from PB, SPB, and STPB-3 in PBS at pH 5.5; Table S3: Kinetic-model fitting parameters for 5-FU release from PB, SPB, and STPB-3 in PBS at pH 7.4 containing glucose (4 mg/mL).

## Abbreviations

The following abbreviations are used in this manuscript:

ABTS	2,2′-Azino-bis(3-ethylbenzothiazoline-6-sulfonic acid)
CFU	Colony-forming unit
DMEM	Dulbecco’s modified Eagle medium
DMSO	Dimethyl sulfoxide
DPPH	2,2-Diphenyl-1-picrylhydrazyl
DTG	Derivative thermogravimetry
FBS	Fetal bovine serum
FE-SEM	Field-emission scanning electron microscopy
FTIR	Fourier transform infrared spectroscopy
5-FU	5-Fluorouracil
GMS	Glutamate–Mannitol–Salts
GPC	Gel permeation chromatography
HEK-293	Human embryonic kidney 293
MTT	3-(4,5-Dimethylthiazol-2-yl)-2,5-diphenyltetrazolium bromide
MWCO	Molecular weight cut-off
NMR	Nuclear magnetic resonance
PB	Poly(vinyl alcohol)/borax hydrogel
PBS	Phosphate-buffered saline
PTIO	2-Phenyl-4,4,5,5-tetramethylimidazoline-1-oxyl 3-oxide
PVA	Poly(vinyl alcohol)
SG	Succinoglycan
SG-TA	Tannic acid-grafted succinoglycan
SPB	Succinoglycan/poly(vinyl alcohol)/borax hydrogel
STPB	Tannic acid-grafted succinoglycan/poly(vinyl alcohol)/borax hydrogel
TA	Tannic acid
TAE	Tannic acid equivalent
TGA	Thermogravimetric analysis
XRD	X-ray diffraction
