# Supplementary material for: Intrinsically Bioactive Tannic Acid-Grafted Succinoglycan for Self-Healing and Stimuli-Responsive Drug Delivery Hydrogels
Source: Gels. 2026 Sep 10;12(9):828. doi: 10.3390/gels12090828 (PMC13606900; doi:10.3390/gels12090828)
Supplement: Supplementary file 1 [file gels-12-00828-s001.zip › gels-4516518-supplementary.pdf]

Supplementary Information

Article

Intrinsically Bioactive Tannic Acid-Grafted Succinoglycan for Self-Healing and Stimuli-Responsive Drug Delivery Hydrogels

Sang-il Park [1,†], Kyungho Kim [1,†], Sungmin Rhyu [1] and Seunho Jung [1,2,*]

[1] Department of Bioscience and Biotechnology, Microbial Carbohydrate Resource Bank (MCRB), Konkuk University, 120 Neungdong-ro, Gwangjin-gu, Seoul 05029, Republic of Korea; qkrtkddlf503@naver.com (S.-I.P.); rudgh971225@naver.com (K.K.); rsm127@naver.com (S.R.)

[2] Department of System Biotechnology, Konkuk University, 120 Neungdong-ro, Gwangjin-gu, Seoul 05029, Republic of Korea

[*] Correspondence: shjung@konkuk.ac.kr

[†] These authors contributed equally to this work.

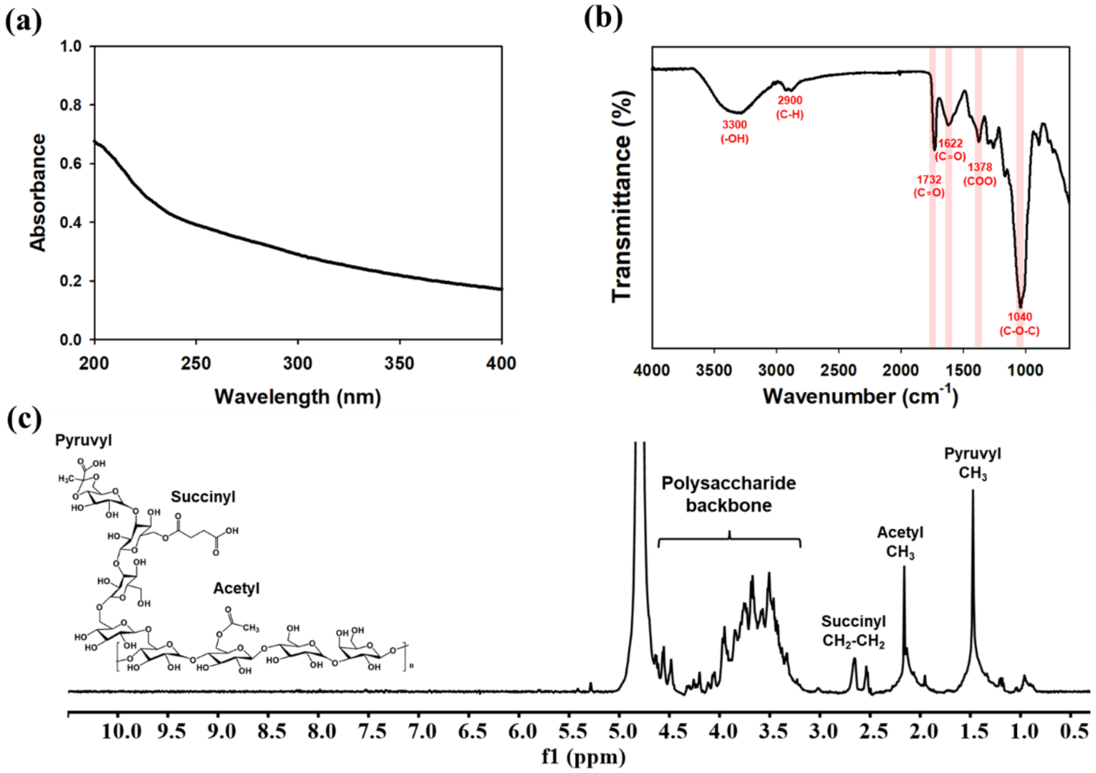

Figure S1. Additional characterization of purified SG: (a) UV–Vis absorbance spectrum, (b) FTIR spectrum, and (c) ^{1}H NMR spectrum.

SG

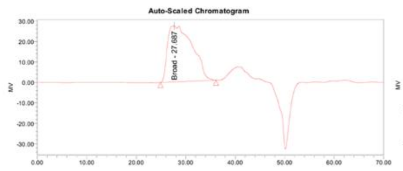

Mn	Mw	MP	Polydispersity
238286	324264		1.360821

SG-TA1

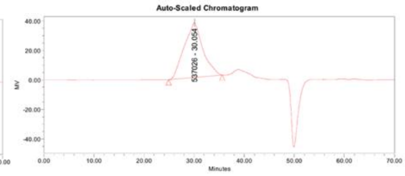

Mn	Mw	MP	Polydispersity
256343	348605	537026	1.359917

SG-TA2

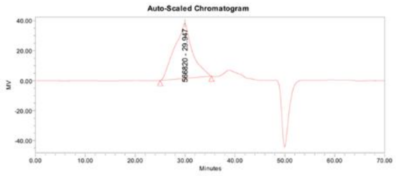

Mn	Mw	MP	Polydispersity
265084	360625	566820	1.360416

SG-TA3

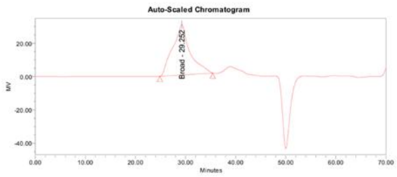

Mn	Mw	MP	Polydispersity
247034	352775		1.428042

SG-TA4

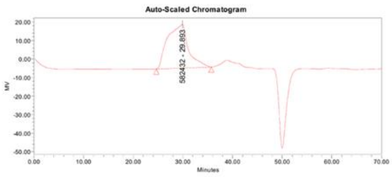

Mn	Mw	MP	Polydispersity
273408	379066	582432	1.386446

SG-TA5

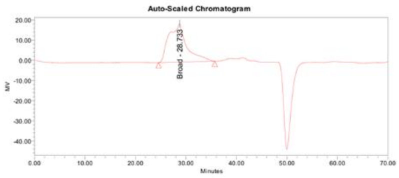

Mn	Mw	MP	Polydispersity
229921	336283		1.462599

Figure S2. GPC chromatograms of SG and SG-TA1–SG-TA5. The corresponding number-average molecular weight (Mn), weight-average molecular weight (Mw), and polydispersity index (PDI) are shown for each sample.

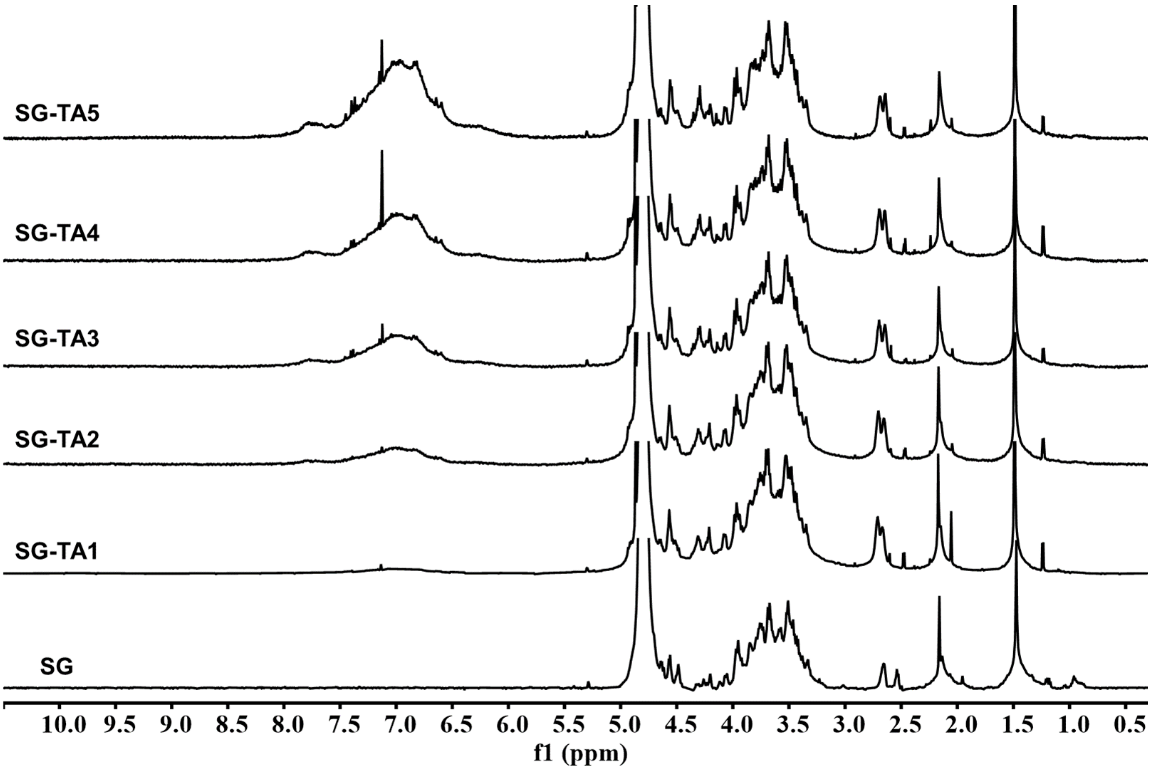

Figure S3. ^{1}H NMR spectra of SG and SG-TA derivatives prepared with different tannic acid feed ratios.

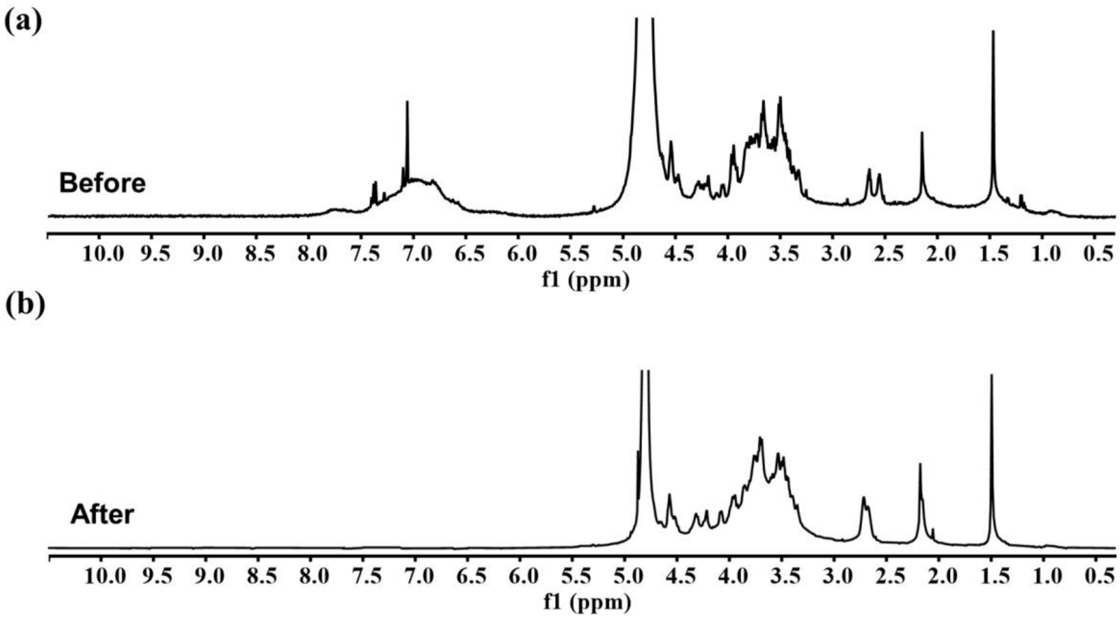

Figure S4. ^1H NMR spectra of the SG/tannic acid physical mixture (a) before and (b) after dialysis.

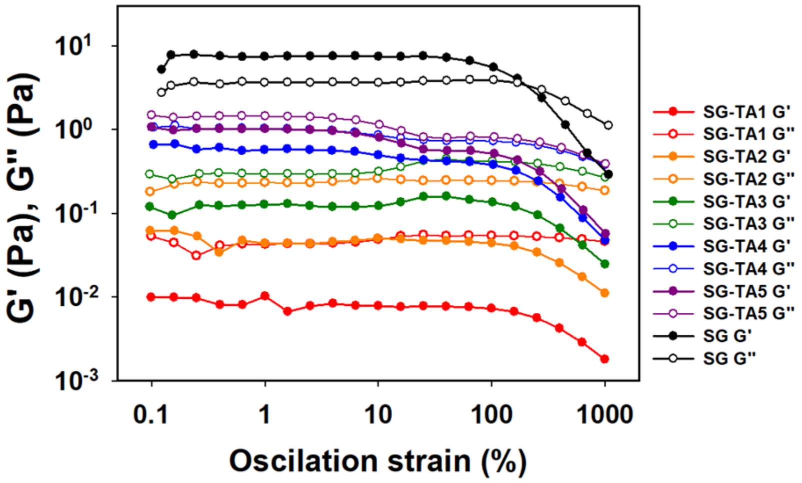

Figure S5. Amplitude-sweep rheological profiles of SG and SG-TA1–SG-TA5 solutions at 25 °C, showing storage modulus (G′) and loss modulus (G″) as a function of strain (0.1–1000%) at an angular frequency of 1.0 rad/s.

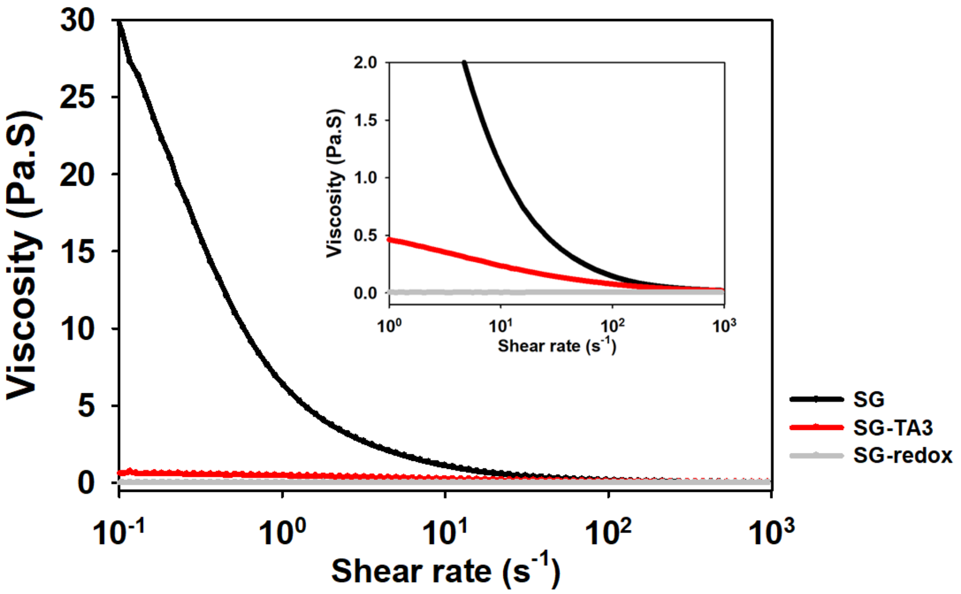

Figure S6. Effect of H_2O_2/ascorbic acid redox treatment on the shear viscosity of SG. Shear-viscosity profiles of 1% (w/v) untreated SG, SG-TA3, and SG-redox were compared over a shear-rate range of 0.1–1000 s^{-1} at 25 °C. SG-redox denotes SG subjected to the same H_2O_2/ascorbic acid treatment and purification procedure as SG-TA, but without tannic acid. The inset shows an enlarged view of the low-viscosity region.

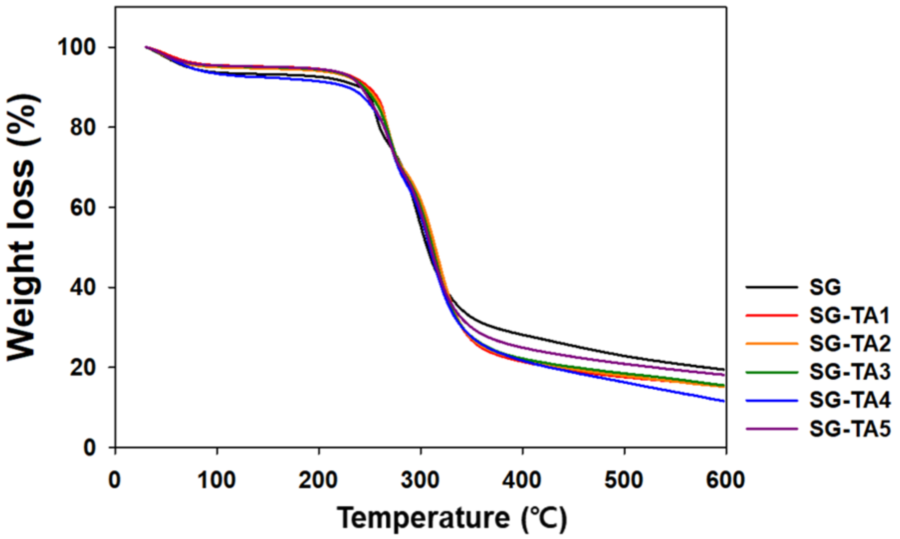

Figure S7. TGA curves of SG and SG-TA1–SG-TA5 recorded under N_2 from 30 to 600 °C at a heating rate of 10 °C min^{-1}.

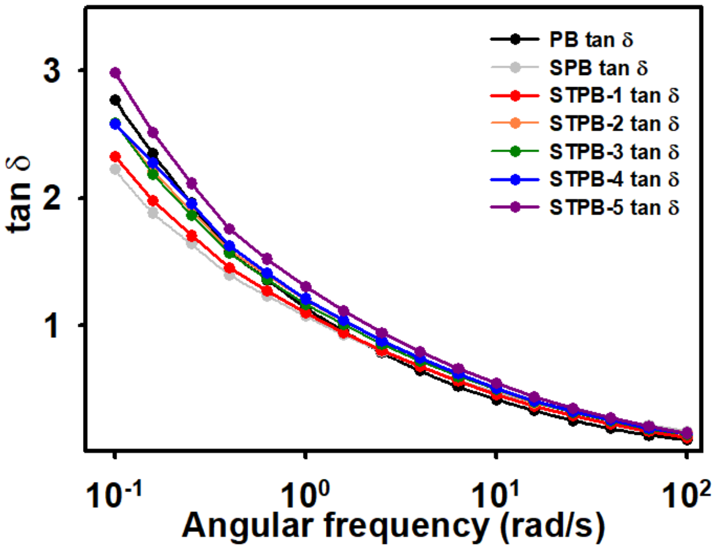

Figure S8. Frequency-dependent loss tangent (tan δ = G″/G′) of PB, SPB, and STPB hydrogels measured over an angular-frequency range of 0.01–100 rad/s at 0.5% strain and 25 °C.

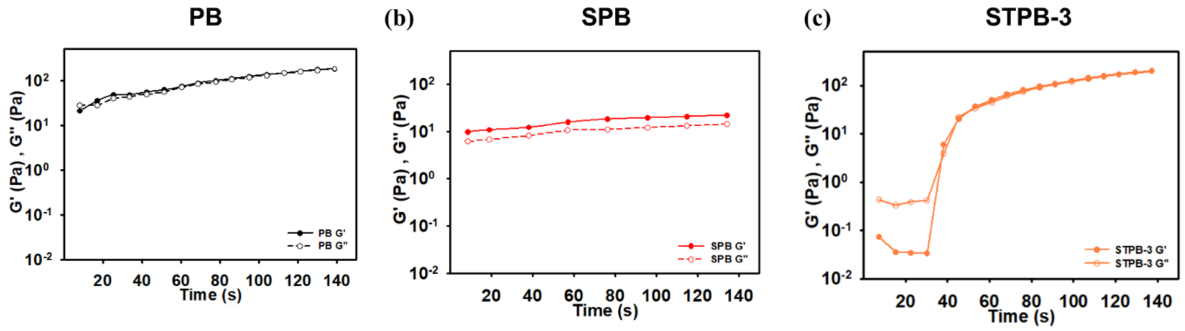

Figure S9. Time-sweep rheological profiles of freshly prepared (a) PB, (b) SPB, and (c) STPB-3 hydrogels, showing the time-dependent evolution of storage modulus (G′) and loss modulus (G″) during network formation.

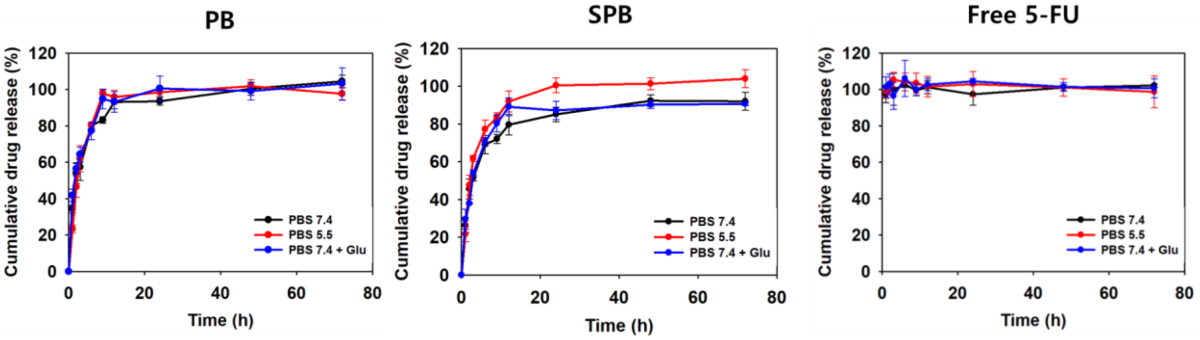

Figure S10. 5-FU release profiles of control formulations under different release conditions: (a) 5-FU-loaded PB, (b) 5-FU-loaded SPB, and (c) free 5-FU in PBS at pH 7.4, PBS at pH 5.5, and PBS at pH 7.4 containing glucose (4 mg/mL)

at 37 °C. Data are presented as mean ± SD (n = 3).

Table S1. Kinetic-model fitting parameters for 5-FU release from PB, SPB, and STPB-3 in PBS at pH 7.4.

	**Model**	**C_0**	**k**	**n**	**R^2**
**PB**	Zero-order	63.568	0.711	–	0.536
	First-order	48.720	0.100	–	0.791
	Higuchi	–	7.876	–	0.721
	Korsmeyer–Peppas	–	35.266	0.487	0.932
**SPB**	Zero-order	53.364	0.665	–	0.550
	First-order	44.842	0.026	–	0.773
	Higuchi	–	7.370	–	0.740
	Korsmeyer–Peppas	–	26.493	0.628	0.959
**STPB-3**	Zero-order	59.706	0.523	–	0.398
	First-order	36.088	0.019	–	0.539
	Higuchi	–	6.084	–	0.589
	Korsmeyer–Peppas	–	30.525	0.584	0.961

C_0, model-derived initial release parameter; k, release rate constant; n, release exponent of the Korsmeyer–Peppas model; R^2, coefficient of determination. Korsmeyer–Peppas fitting was performed only when sufficient data points were available within the initial release region ($M_t/M_\infty \leq 0.6$).

Table S2. Kinetic-model fitting parameters for 5-FU release from PB, SPB, and STPB-3 in PBS at pH 5.5.

	**Model**	**C_0**	**k**	**n**	**R^2**
**PB**	Zero-order	66.217	0.720	–	0.372
	First-order	119.794	0.422	–	0.841
	Higuchi	–	8.484	–	0.566
	Korsmeyer–Peppas	–	–	–	–
**SPB**	Zero-order	61.070	0.820	–	0.500
	First-order	80.162	0.194	–	0.975
	Higuchi	–	9.207	–	0.691
	Korsmeyer–Peppas	–	–	–	–
**STPB-3**	Zero-order	68.179	0.715	–	0.422
	First-order	79.390	0.253	–	0.982
	Higuchi	–	8.246	–	0.615

	Korsmeyer–Peppas	–	–	–	–

C_0, model-derived initial release parameter; k, release rate constant; n, release exponent of the Korsmeyer–Peppas model; R^2, coefficient of determination. Korsmeyer–Peppas fitting was performed only when sufficient data points were available within the initial release region ($M_t/M_\infty \leq 0.6$).

Table S3. Kinetic-model fitting parameters for 5-FU release from PB, SPB, and STPB-3 in PBS at pH 7.4 containing glucose (4 mg/mL).

	**Model**	**C_0**	**k**	**n**	**R^2**
**PB**	Zero-order	68.703	0.618	–	0.470
	First-order	33.307	0.075	–	0.792
	Higuchi	–	7.020	–	0.665
	Korsmeyer–Peppas	–	–	–	–
**SPB**	Zero-order	56.129	0.614	–	0.425
	First-order	38.615	0.022	–	0.552
	Higuchi	–	7.085	–	0.621
	Korsmeyer–Peppas	–	27.914	0.528	0.940
**STPB-3**	Zero-order	67.821	0.628	–	0.450
	First-order	54.097	0.168	–	0.875
	Higuchi	–	7.184	–	0.646
	Korsmeyer–Peppas	–	–	–	–

C_0, model-derived initial release parameter; k, release rate constant; n, release exponent of the Korsmeyer–Peppas model; R^2, coefficient of determination. Korsmeyer–Peppas fitting was performed only when sufficient data points were available within the initial release region ($M_t/M_\infty \leq 0.6$).
